# An Insect Counteradaptation against Host Plant Defenses Evolved through Concerted Neofunctionalization

**DOI:** 10.1093/molbev/msz019

**Published:** 2019-02-01

**Authors:** Hanna M Heidel-Fischer, Roy Kirsch, Michael Reichelt, Seung-Joon Ahn, Natalie Wielsch, Simon W Baxter, David G Heckel, Heiko Vogel, Juergen Kroymann

**Affiliations:** 1 Department of Entomology, Max Planck Institute for Chemical Ecology, Jena, Germany; 2 Department of Biochemistry, Max Planck Institute for Chemical Ecology, Jena, Germany; 3 Mass Spectrometry Group, Max Planck Institute for Chemical Ecology, Jena, Germany; 4 Molecular and Biomedical Science, School of Biological Sciences, University of Adelaide, Adelaide, Australia; 5 Génétique et Écologie Évolutives, Écologie Systématique Evolution, CNRS/Université Paris-Sud/AgroParisTech, Université Paris-Saclay, Orsay Cedex, France; 6 Department of Crop and Soil Science, Oregon State University, Corvallis, OR

**Keywords:** chemical ecology, molecular evolution, plant–insect interactions, insect counteradaptation, *Plutella xylostella*, glucosinolate–myrosinase complex, glucosinolate sulfatase, coevolutionary arms race, neofunctionalization, escape from adaptive conflict, concerted neofunctionalization

## Abstract

Antagonistic chemical interactions between herbivorous insects and their host plants are often thought to coevolve in a stepwise process, with an evolutionary innovation on one side being countered by a corresponding advance on the other. Glucosinolate sulfatase (GSS) enzyme activity is essential for the Diamondback moth, *Plutella xylostella*, to overcome a highly diversified secondary metabolite-based host defense system in the Brassicales. *GSS* genes are located in an ancient cluster of arylsulfataselike genes, but the exact roles of gene copies and their evolutionary trajectories are unknown. Here, we combine a functional investigation of duplicated insect arylsulfatases with an analysis of associated nucleotide substitution patterns. We show that the Diamondback moth genome encodes three GSSs with distinct substrate spectra and distinct expression patterns in response to glucosinolates. Contrary to our expectations, early functional diversification of gene copies was not indicative of a coevolutionary arms race between host and herbivore. Instead, both copies of a duplicated arylsulfatase gene evolved concertedly in the context of an insect host shift to acquire novel detoxifying functions under positive selection, a pattern of duplicate gene retention that we call “concerted neofunctionalization.”

## Introduction

**Fig. 1.**
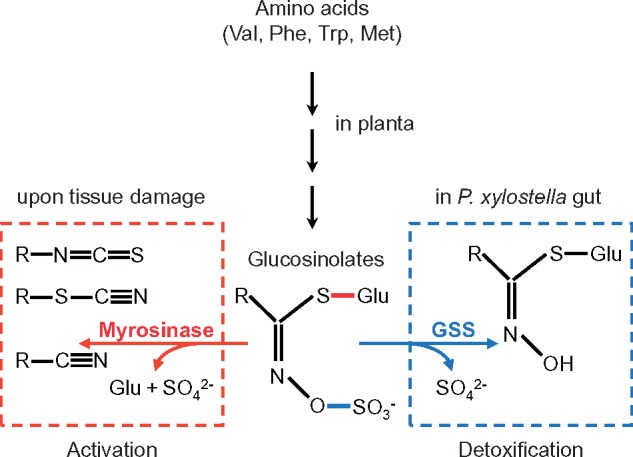
GS activation and detoxification. Amino acid–derived plant GS can be activated by myrosinases (left) via hydrolysis of the thioglucose ester bond (red) upon plant tissue damage to yield toxic products or are detoxified in *Plutella xylostella* guts (right) by GSS-mediated removal of the sulfate group (blue).

Adaptation of a herbivore to a new host plant requires evolutionary innovation to circumvent host defenses (Ehrlich and Raven 1964; Berenbaum and Feeny 1981; Li et al. 2003). More than 20 Ma (Sohn et al. 2013; Wahlberg et al. 2013) ermine moths shifted toward host plants in the order Brassicales, which have existed for about 100 My (Edger et al. 2015; Magallón et al. 2015; Tank et al. 2015; Cardinal-McTeague et al. 2016). These plants possess an activated chemical defense system, the glucosinolate (GS)–myrosinase complex, which protects against herbivorous insects and other enemies (Kliebenstein et al. 2005). Amino acid–derived GSs (β-d-thioglucoside-*N*-hydroxysulfates) are compartmentalized separate from myrosinases, which can hydrolyze GS. When tissue damage brings myrosinases into contact with GS, they remove the β-d-thioglucoside moiety, and the aglycones degrade rapidly to toxic substances (fig. 1 ). The capacity to synthesize GS from Phe or Trp is widespread in the Brassicales, whereas biosynthesis of GS from Met is an evolutionary innovation in the youngest families of this plant order. Emergence of Met-GS greatly increased GS chemical diversity (Agerbirk and Olsen 2012) and is thought to account at least partly for the evolutionary success of the Brassicaceae (Edger et al. 2015; Cardinal-McTeague et al. 2016), which include the model plant *Arabidopsis thaliana* and several important cruciferous vegetables. Phe- and Trp-GS are typically inducible by defense hormone signaling, but salicylic or jasmonic acid have little influence on Met-GS biosynthesis (Bodnaryk 1994; Doughty et al. 1995; Brader et al. 2001; Ludwig-Müller et al. 2002; Mikkelsen et al. 2003; Textor and Gershenzon 2009).

**Fig. 2.**
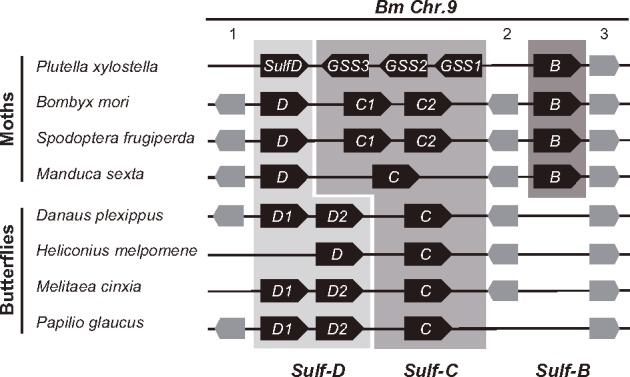
*GSS* genes are located in an ancient arylsulfatase gene cluster. This gene cluster consists principally of *Sulf-D*, *-C*, and *-B* genes, with lineage-specific duplications and deletions. In the *Bombyx mori* (*Bm*) genome, this cluster is located on Chromosome 9. Intervening genes code for inositol polyphosphate-1-phosphatase (1), an olfactory receptor (2), and fasciclin-1 (3). Note the inversion of *GSS1–3* relative to flanking arylsulfatases.

*Plutella xylostella* (Diamondback moth [DBM]) is one of the most destructive pests on cruciferous vegetables, and the insect has evolved resistance against multiple pesticides (Shelton et al. 1993; Talekar and Shelton 1993; Furlong et al. 2013). Glucosinolate sulfatase (GSS) activity is the central counteradaptation of DBM against the plant GS–myrosinase complex (Ratzka et al. 2002). GSS removes the sulfate group of GSs; the desulfated forms cannot be hydrolyzed by myrosinase and are excreted with the feces (fig. 1). Originally, GSS activity had been attributed to a single gene product, *Px*GSS1, but sequencing of the DBM genome (You et al. 2013) revealed that this gene is part of a cluster of several arylsulfataselike genes. This gene cluster is also present in other moths and butterflies and consists principally of three genes, termed *SulfD*, *C*, and *B*, but has been subject to lineage-specific gene duplications and losses (fig. 2). Compared with other lepidopterans, the central part of the DBM arylsulfatase gene cluster is inverted, comprising *PxGSS1* and two other arylsulfataselike genes (termed *PxGSS2* and *PxGSS3*). *Px*GSS1 shares 95% and 65% amino acid identity with the translated open reading frames of *PxGSS2* and *PxGSS3*, respectively, but <40% identity with the arylsulfataselike genes flanking either side of the inversion, *PxSulfB* and *PxSulfD*, suggesting that *PxGSS2* and *PxGSS3* may also encode GSS activity.

Gene duplication plays an important role for evolutionary innovation (Ohno 1970) and occurs at a high rate, but typically, one copy of a duplicate gene pair degenerates and eventually disappears (Lynch and Conery 2000). Duplicate genes can be stably retained in the genome when they subdivide ancestral gene functions by complementary deleterious mutations, a process called subfunctionalization (SF) (Force et al. 1999), when they evolve a new adaptive function (Ohno 1970), or in combination of both processes (He and Zhang 2005). Two alternative models explain retention and divergence of duplicate genes under positive selection, neofunctionalization (Ohno 1970) (NF) and escape from adaptive conflict (EAC) (Ohno 1970; Piatigorsky and Wistow 1991; Hughes 1994; Des Marais and Rausher 2008; Innan and Kondrashov 2010). Both models are supported by case studies (Piatigorsky and Wistow 1991; Zhang et al. 2002; Benderoth et al. 2006; Hittinger and Carroll 2007; Des Marais and Rausher 2008; Fucile et al. 2008; Storz et al. 2008; Deng et al. 2010; Huang et al. 2015). In the NF model, one gene copy acquires a new, beneficial activity after gene duplication, whereas the other preserves the ancestral function. NF is associated with positive selection on the new function and purifying selection on the ancestral function. In the EAC model, an adaptive conflict arises before gene duplication, when a single-copy gene evolves a novel function in addition to maintaining its ancestral role. Antagonistic pleiotropy moves the ancestral gene function away from its previous local adaptive optimum and prevents the new function from reaching its own adaptive optimum. Gene duplication can resolve this conflict, such that one copy restores optimal ancestral function, whereas the other copy improves the novel function. Hence, both copies evolve under positive selection but the strength of selection on the ancestral function depends on how far this function had shifted away from its optimum after emergence of the novel function. To distinguish between NF and EAC, it is necessary to analyze both evolutionary trajectories of duplicate genes and functional properties of encoded gene products (Des Marais and Rausher 2008).

In this work, we combine functional and evolutionary analyses of DBM arylsulfataselike genes and enzymes to gain insight into emergence and evolution of an insect counteradaptation against host plant chemical defenses. We show that the genetic architecture of the DBM counteradaptation is more complex than previously thought (Ratzka et al. 2002); it consists of three tandemly arranged arylsulfataselike genes that encode GSS activity and evolved from an ancestral *SulfC* gene. *PxGSS1*, *2*, and *3* differ in their response to GS and the encoded enzymes detoxify different spectra of GS. Early functional divergence of *GSS* genes is not explained by an expansion of GSS substrate range upon gene duplication in response to evolutionary innovations in host plant GS-based chemical defense. Instead, we infer that *GSS* genes diverged concertedly in the process of an insect host shift, marked by traces of positive selection in both daughter genes of the initial *SulfC* duplication in the *Plutella* lineage.

## Results

To test our hypothesis that the DBM genome encoded several GSSs, we first investigated whether *PxGSS1*, *2*, and *3*, and *PxSulfD* were expressed in larval guts, the organ where GSS activity is located (Ratzka et al. 2002). We examined four DBM strains from three continents: North American G88, a South Australian strain collected from oilseed rape (DBM-R, also known as Waite), and two African strains collected in Kenya from cabbage (DBM-C) and pea (DBM-P) (Shelton et al. 1993; Henniges-Janssen, Reineke, et al. 2011; Henniges-Janssen, Schöfl, et al. 2011). We separated guts from the rest of the bodies of larvae reared on standard artificial diet or their respective host plants. Real-time quantitative polymerase chain reaction (PCR) revealed that *PxGSS1*, *2*, and *3* transcripts were highly abundant in the gut of all strains, whereas only minute amounts were detectable in the rest of the larval body (supplementary fig. S1, Supplementary Material online). By contrast, *PxSulfD* transcripts had low abundance in both gut and larval body. Peptide sequencing by mass spectrometry (MS^E^) of DBM-R confirmed that the corresponding proteins, *Px*GSS1, *Px*GSS2, and *Px*GSS3, were present in guts (supplementary fig. S2, Supplementary Material online).

**Fig. 3.**
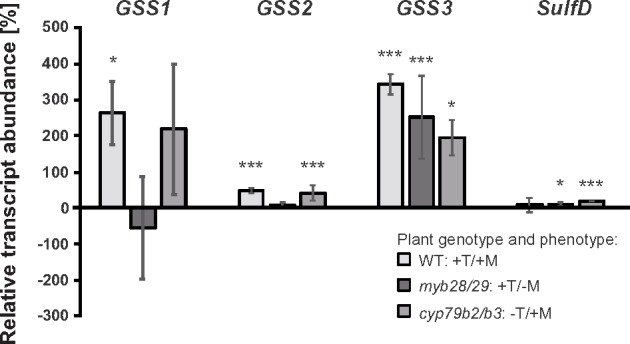
GS-dependent transcription of arylsulfatase genes from *Plutella xylostella*. DBM-C larvae fed on four Arabidopsis genotypes with distinct GS phenotypes. Arabidopsis wildtype (WT) has both Trp- and Met-derived GS (+T/+M), whereas *myb28/29*, *cyp79b2/b3*, and *cyp79b2/b3/myb28/29* mutants are defective in Met-GS (+T/−M), Trp-GS (−T/+M), and Met- and Trp-derived GS (null mutant), respectively. Shown are relative differences in arylsulfatase transcript abundance (±SEM of three biological replicates) in comparison to the GS null mutant (*P ≤* *0.05 and ***P ≤* *0.001), according to post hoc two-tailed *t*-tests.

**Fig. 4.**
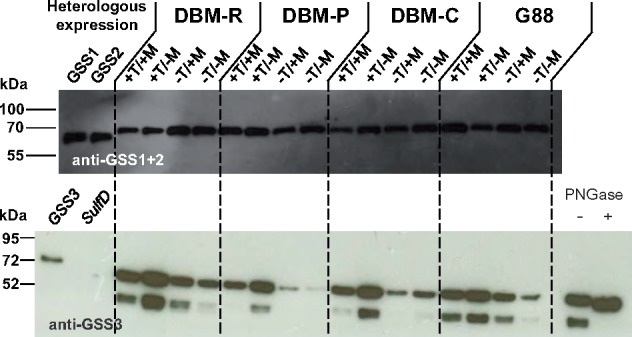
GS-dependent expression of arylsulfatase genes from *Plutella xylostella*. Larval gut protein was extracted from four *P. xylostella* strains, DBM-R, -P, -C, and G88, after herbivory on Arabidopsis wildtype (+T/+M) or mutants impaired in GS biosynthesis, *myb28/29* (+T/−M), *cyp79b2/b3* (−T/+M) and *cyp79b2/b3/myb28/29* (−T/-M); T = Trp-GS, M = Met-GS. Western blot hybridization was conducted with anti-GSS1/2 (upper panel) or anti-GSS3 (lower panel). Recombinant GSS1 and GSS2, and GSS3 and SulfD were included as controls. Prior to gel loading all samples in the upper panel were treated with PNGase F. In the lower panel, only one sample (right) was treated with PNGase F, showing that two bands detected with anti-GSS3 merged into a single band upon removal of *N*-linked oligosaccharides. Upon hybridization with anti-GSS1 + 2, samples showed only minor variation in hybridization intensity. By contrast, GSS3 protein abundance was strongly dependent on plant GS profiles. In particular, presence of Trp-GS in the absence of Met-GS (*myb28/29*) led to high GSS3 abundance whereas absence of Trp-GS (*cyp79b2/b3* and *cyp79b2/b3/myb28/29*) resulted in lowered GSS3 abundance compared with Arabidopsis wildtype (Col-0).

Next, we investigated whether gene expression patterns were influenced by GS in DBM-C larvae reared on a variety of *A. thaliana* mutants with specific defects in GS biosynthesis. Mutants in *cyp79b2/b3*, *myb28/29*, and *cyp79b2/b3/myb28/2*9 are impaired in Trp-, Met-, or Met- and Trp-GS, respectively (Zhao et al. 2002; Sønderby et al. 2007; Sun et al. 2009; Müller et al. 2010), and their common genetic background, Col-0, does not contain foliar Phe-GS (Kliebenstein et al. 2001). *PxGSS1*, *2*, and *3* transcripts were less abundant in guts from larvae reared on mutant plants (fig. 3), indicating that expression of these genes was partly GS dependent. *PxGSS1* and *2* transcript levels were inducible by Met-GS but did not respond significantly to Trp-GS, whereas Trp-GS clearly induced the level of *PxGSS3* transcripts. By contrast, host plant genotype had little influence on *PxSulfD* expression. To confirm these findings at the protein level, we conducted Western blot hybridization of protein extracts from larval guts separated by sodium dodecyl sulfate polyacrylamide gel electrophoresis. For hybridization, we used a polyclonal rabbit GSS1/2-antibody (Ratzka et al. 2002) that detected both *Px*GSS1 and *Px*GSS2 with similar efficiency, and a new polyclonal rabbit antibody, raised against heterologously expressed *PxGSS3*. Hybridization with the GSS1/2 antibody yielded a nearly uniform pattern for *Px*GSS1 + *Px*GSS2 protein abundance in the guts of larvae from all four strains, independent of the food source (fig. 4). By contrast, *Px*GSS3 quantity was strongly affected by *A. thaliana* Trp-GS content; compared with wildtype plants, the GSS3 antibody detected more *Px*GSS3 protein when G88, DBM-C, -R, or -P larvae had fed on Trp GS–producing *myb28/29*, and less GSS3 when larvae were reared on Trp GS–lacking *cyp79b2*/*b3* or *cyp79b2*/*b3*/*myb28*/*29*.

Taken together, expression patterns suggested that *Px*GSS1, 2, and 3 (but not *Px*SulfD) could participate in GS detoxification, with *Px*GSS3 being particularly important for detoxification of Phe-/Trp-GS and *Px*GSS1 and 2 for Met-GS. To confirm these hypotheses, we expressed *PxGSS1*, *2*, and *3* in *Sf*9 insect cells, an expression system that properly processes arylsulfatases (Ratzka et al. 2002). We also tested recombinant *Bombyx mori* C1 (*Bm*C1), *Yponomeuta cagnagella* C (*Yc*C), and *Px*SulfD to evaluate whether other arylsulfatases might have cryptic GSS activity. All enzymes cleaved the arylsulfatase standard substrate 4-methylumbelliferyl sulfate (Sherman and Stanfield 1967) (supplementary fig. S3, Supplementary Material online, and table 1), showing that recombinant enzymes were functional arylsulfatases. Next, we tested a variety of substrates representative of different GS classes. Assays showed strikingly different spectra of substrate specificities for the different enzymes (table 1 and supplementary fig. S4, Supplementary Material online): *Px*GSS3 accepted Phe-, Trp-, and Val-GS as substrates but no Met-GS. *Px*GSS1 desulfated all GSs, except for 1MOI3M, a Trp-GS that is typically abundant in plant roots but rare in leaves (Pfalz et al. 2016). *Px*GSS2 accepted only a subset of Met-GS and no Phe- or Trp-GS as substrates. Finally, *Px*SulfD, *Bm*C1, and *Yc*C did not hydrolyze any of these GS (supplementary figs. S4 and S5, Supplementary Material online). Hence, the present-day DBM genome indeed encodes three GSS, which evolved from an arylsulfatase of as yet unknown function.

**Table 1.** Activity of Insect Arylsulfatases with GSs in HPLC-Based Assays

Enzyme	Substrate											
	Val-GS	Phe-GS	Trp-GS			Met-GS						
	1-ME	pOHB	I3M	4MOI3M	1MOI3M	Sinigrin	4-MTB	4-MSOB	9-MSON	10-MSOD	4-Pent	4-MLF
*Px*GSS1	+	+	+	+	/	+	+	+	+	+	+	+
*Px*GSS2	(+)	/	/	/	/	/	(+)	/	+	+	/	+
*Px*GSS3	(+)	+	+	+	+	/	/	/	/	/	/	+
*Bm*C1	/	/	/	/	/	/	/	/	/	/	/	+
*Yc*C	/	/	/	/	/	/	/	/	/	/	/	+
*Px*SulfD	/	/	/	/	/	/	/	/	/	/	/	+

1-ME, 1-methylethyl GS; 4-MTB, 4-methylthiobutyl GS; 4-MSOB, 4-methylsulfinylbutyl GS; 9-MSON, 9-methylsulfinylnonyl GS; 10-MSOD, 10-methylsulfinyldecyl GS; 4-Pent, 4-pentenyl GS; pOHB, *p*-hydroxybenzyl GS; I3M, indol-3-ylmethyl GS; 4MOI3M, 4-methoxyindol-3-ylmethyl GS; 1MOI3M, 1-methoxyindol-3-ylmethyl GS; 4-MLF, 4-methylumbelliferyl sulfate; +, activity; (+), weak activity; /, not detected.

**Fig. 5.**
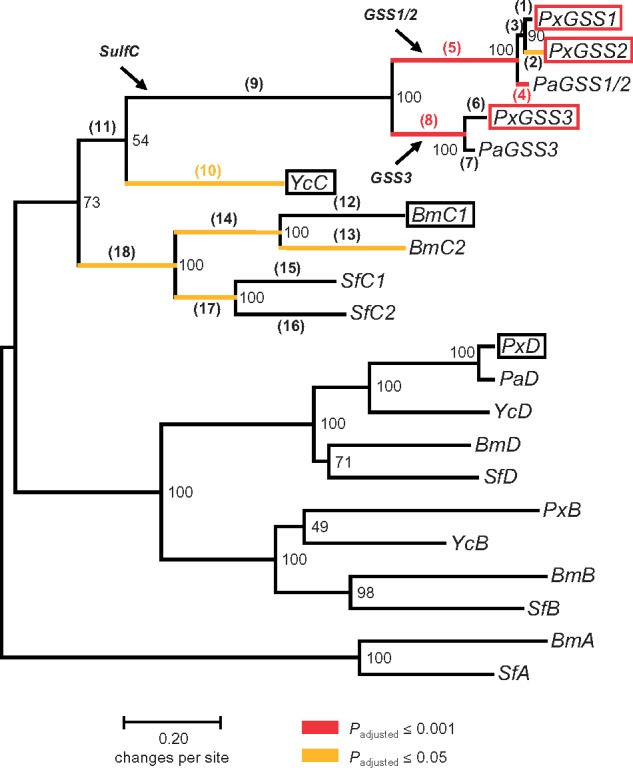
Maximum-likelihood tree of moth arylsulfatase genes. *Px*, *Plutella xylostella*; *Pa*, *Plutella australiana*; *Yc*, *Yponomeuta cagnagella*; *Bm*, *Bombyx mori*; *Sf*, *Spodoptera frugiperda*. *GSS*: GS sulfatase gene, *A*, *B*, *C*, and *D*: *SulfA-*, *B-*, *C-*, and *D*-type arylsulfatase genes. *PxGSS1*, *2*, and *3* are represented by two alleles each. *SulfC*, *GSS1/2*, and *GSS3* indicate genes before and after the initial *SulfC* duplication in the *Plutella* lineage, respectively. *SulfA* sequences were used as the outgroup. Numbers next to nodes indicate percentage of bootstrap support (1,000 replicates). The tree is drawn to scale, with branch lengths measured as the number of substitutions per site. Boxes indicate recombinant genes that were tested for GSS activity (black: no GSS activity; red: GSS activity), numbers in brackets show branches that were tested for positive selection. Colored branches indicate branches with evidence for positive selection (orange: *P*_adjusted_ ≤ 0.05; red: *P*_adjusted_ ≤ 0.001). Note that strong positive selection on *GSS* genes caused low bootstrap support for deep branches in the *SulfC* clade.

To determine relationships among *GSS* genes, we constructed a gene tree of lepidopteran arylsulfataselike sequences. We included sequence data from *Plutella australiana*, a close relative of cosmopolitan DBM, but with a distribution limited to Australia (Landry and Hebert 2013; Perry et al. 2018). We identified *P. australiana* orthologs of *PxGSS3* and *PxSulfD* (termed *PaGSS3* and *PaSulfD*). A single *P. australiana* gene, termed *PaGSS1/2*, clustered with *PxGSS1* and *PxGSS2* (fig. 5). All DBM genes coding for enzymes with GSS activity, *PxGSS1*, *PxGSS2*, and *PxGSS3*, as well as their counterparts from *P. australiana*, grouped together in the arylsulfatase gene tree, and this group was most closely related to *SulfC*-type sequences from other species, as expected from their physical position in the arylsulfatase gene cluster (fig. 2).

The gene branching order indicated that the initial *SulfC* duplication, resulting in *GSS3* and *GSS1/2*, occurred early in the *Plutella* lineage, whereas the subsequent duplication of *GSS1/2*, leading to *PxGSS1* and *PxGSS2*, likely took place after the *P. xylostella* and *P. australiana* lineages diverged from a common ancestorabout two Ma (Ward and Baxter 2018). Because all three *GSS* genes in the DBM genome, *PxGSS1*, *PxGSS2*, and *PxGSS3*, coded for enzymes with GSS activity, we deduced that this activity had emerged before *SulfC* duplicated in the *Plutella* lineage. Similarly, absence of cryptic GSS activity in other arylsulfatases suggested that the enzyme encoded by the ancestral *SulfC* gene did likely not possess GSS activity. In other words, the *Plutella* lineage acquired GSS activity after separation from a common ancestor shared with *Yponomeuta*, but before the initial *SulfC* gene duplication took place in the *Plutella* lineage (i.e., GSS activity emerged along branch 9 in fig. 5).

Furthermore, a comparison between DBM GSS substrate spectra and GS distribution in the Brassicales led us to hypothesize that different DBM GSS functions had been established stepwise in a coevolutionary arms race, to adapt to evolutionary changes in host plant defense metabolite composition. Under this hypothesis, GSS3-like activity against Phe- and Trp-GS would have evolved first to overcome these phylogenetically ancient classes of GS. GSS1-like activity, with a broader substrate spectrum, would have countered the later emergence of Met-GS as novel plant defense metabolites in the Brassicales. Finally, GSS2-like activity would be a subsequent specialization to detoxify long-chain Met-GS.

GS hydrolysis products are lethal to DBM (Li et al. 2000; Morimoto et al. 2000). We therefore expected that establishment of the GSS function (fig. 5, branch 9), expansion of the GSS activity spectrum to include Met-GS (fig. 5, branch 5), and possibly also subsequent specialization toward long-chain Met-GS (fig. 5, branch 2) were subject to episodic positive selection in the past. We tested these hypotheses by inspecting all branches of the *SulfC* clade in the gene tree with branch-site codon substitution models (Yang and Nielsen 2002; Zhang et al. 2005).

There was some evidence for positive selection prior to the *SulfC* gene duplication in the *Plutella* lineage (fig. 5 and supplementary tables S1–S3, Supplementary Material online). A model allowing for positive selection along branch 9 (fig. 5) explained the data better than a nearly neutral model (*Χ^2^_1_* = 6.7; *P*_unadjusted_ = 0.010), but this evidence was not supported upon correction for multiple testing. By contrast, there was strong statistical support for positive selection subsequent to this gene duplication. But contrary to our expectations, both daughter branches, the *GSS3*-branch (branch 8; *Χ^2^_1_* = 34.7; *P*_adjusted_ = 0.000) and the *GSS1/2*-branch (branch 5; *Χ^2^_1_* = 33.1; *P*_adjusted_ = 0.000), had evolved under positive selection. The second gene duplication, leading to *PxGSS1* and *PxGSS2*, however, displayed the expected signatures typical for NF, with positive selection on *PxGSS2* (branch 2; *Χ^2^_1_* = 8.2; *P*_adjusted_ ≤ 0.05) and purifying selection on *PxGSS1* (branch 1). Finally, there was evidence for positive selection acting on *PaGSS1/2* (branch 4; *Χ^2^_1_* = 20.2; *P*_adjusted_ < 0.001) after speciation. This could indicate further specialization of *P. australiana* on host plants with a distinct GS profile in Australia, a hypothesis that remains to be tested.

We employed other phylogeny-based divergence tests, BUSTED (Murrell et al. 2015) and aBSREL (Smith et al. 2015). These tests also found evidence for episodic diversifying selection on the *GSS1/2*- and *GSS3*-branches, on *PxGSS2*, and on *PaGSS1/2* (supplementary tables S4 and S5, Supplementary Material online), but there was no indication for positive selection on the *SulfC*-branch before gene duplication.

Importantly, the pattern of nucleotide substitutions post *SulfC* duplication, indicative of positive selection on both copies of a duplicated gene pair, was highly unexpected. Therefore, we employed population-based divergence tests (McDonald and Kreitman 1991) to verify our findings. We sampled *PxGSS1*, *PxGSS2*, and *PxGSS3* sequences from all four DBM strains, G88, DBM-R, DBM-C, and DBM-P. We compared polymorphism within *P. xylostella* with substitutions between *P. xylostella* and reconstructed ancestral sequences to disentangle the shared evolutionary history of *GSS* genes. We used phylogenetic analysis by maximum likelihood (PAML) to reconstruct sequences at key nodes of the arylsulfatase gene tree under a set of different assumptions, including a neutral model and branch-site models that forced positive selection to have occurred before or after *SulfC* duplication. Importantly, the model that forced positive selection to have occurred before *SulfC* gene duplication (i.e., along branch 9 in fig. 5) provided a conservative reference for testing positive selection after *SulfC* duplication. Similarly, models that forced positive selection onto the *GSS1/2*-branch (branch 5) or the *GSS3*-branch (branch 8) provided a conservative reference for testing positive selection on the corresponding sister branches. In addition, we compared polymorphism within *P. xylostella* with substitutions in relation to *Yponomeuta cagnagella SulfC* (*YcC*), to *P. australiana PaGSS1/2* and *PaGSS3*, and we conducted pairwise comparisons between *PxGSS1*, *2*, and *3* (tables 2 and 3 and supplementary table S6, Supplementary Material online).

**Table 2.** McDonald–Kreitman Comparisons between *Plutella xylostella GSS* Genes and Reconstructed Ancestral Sequences.

	*PxGSS1* (*N* = 60)					*PxGSS2* (*N* = 24)					*PxGSS3* (*N* = 40)				
Substitutions	syn	nsyn	**NI**^a^	***χ***2	*P*	syn	nsyn	**NI**^a^	***χ***^2^	*P*	syn	nsyn	**NI**^a^	***χ***^2^	*P*
*YcC*	—	379.6	—	—	—	899.0	378.1	0.33	13.5	0.000	562.8	354.0	0.13	39.6	0.000
*SulfC*^b^^,^^c^	422–508	278–290	≤0.10	≥24.7	0.000	372–445	283–299	≤0.21	≥27.8	0.000	264–394	246–261	≤0.13	≥41.0	0.000
*SulfC*^b^^,^^d^	12–165	97–151	≤0.06	≥34.7	0.000	18–162	100–152	≤0.16	≥36.2	0.000	30–166	55–103	≤0.14	≥34.0	0.000
*GSS1/2*^b^	1–5	5–6	≤0.05	≥20.2	0.000	7–12	11–13	≤0.13	≥18.5	0.000					
*PxGSS1/2*^b^	1	0	—	—	—	5–6	6	0.12	12.8	0.000					
*GSS3*^b^											22–36	5–7	≤0.59	≤4.5	≤0.366
*PaGSS1/2*	17.6	19.2	0.05	28.2	0.000	22.0	23.4	0.13	25.7	0.000					
*PaGSS3*											38.9	10.1	0.32	5.4	0.020
**Polymorphisms**	56	3				86	12				97	8			

a Neutrality Index.

b Reconstructed ancestral sequence.

c Before separation of *Plutella* and *Yponomeuta* lineages.

d Before initial *SulfC* duplication in the *Plutella* lineage.

**Table 3.** McDonald–Kreitman Pairwise Comparisons of *Plutella xylostella GSS* Genes.

	Polymorphisms		Substitutions				
Comparison	syn	nsyn	syn	nsyn	NI	*χ*^2^	*P*
*PxGSS1 vs. PxGSS2*	142.0	15.0	5.1	6.0	0.09	19.0	0.000
*PxGSS1 vs. PxGSS3*	153.0	11.0	168.2	205.9	0.06	110.7	0.000
*PxGSS2 vs. PxGSS3*	183.0	20.0	165.9	207.3	0.09	114.9	0.000

We found an excess of nonsynonymous substitutions for *PxGSS1* (*χ^2^*_JC_ ≥ 34.7; *P *=* *0.000) and *PxGSS2* (*χ^2^*_JC_ ≥ 36.2; *P *=* *0.000) after *SulfC* gene duplication, irrespective of how we had modeled ancestral sequences (table 2 and supplementary table S6, Supplementary Material online). Furthermore, most nonsynonymous substitutions had occurred before the duplication of *GSS1/2* (table 2 and supplementary table S6, Supplementary Material online). This confirmed that *GSS1/2* had evolved under positive selection after *SulfC* duplication. Similarly, we found an excess of nonsynonymous substitutions for *GSS3* (*χ^2^*_JC_ ≥ 34.0; *P *=* *0.000) after *SulfC* gene duplication (table 2 and supplementary table S6, Supplementary Material online) and the comparison of *PxGSS3* and *PaGSS3* indicated that most nonsynonymous substitutions had occurred before separation of the *P. xylostella* and *P. australiana* lineages (table 2 and supplementary table S6, Supplementary Material online). Hence, the tests confirmed that *GSS3* had evolved under positive selection after *SulfC* duplication. We also found an excess of nonsynonymous substitutions for *PxGSS2* (*χ^2^*_JC_ = 12.8; *P *=* *0.000) after *GSS1/2* gene duplication (table 2). Similarly, the comparison between *PxGSS1* and *PxGSS2* indicated an excess of nonsynonymous substitutions (*χ^2^*_JC_ = 19.0; *P *=* *0.000; table 3). Because extant *Px*GSS1 and reconstructed GSS1/2 had identical amino acid sequences, this confirmed that *PxGSS2* had evolved under positive selection after duplication of *GSS1/2*.

## Discussion

The DBM genome encodes three tandemly arranged genes with GSS activity, *PxGSS1*, *2*, and *3*. Two gene duplication events explain this configuration, a duplication of the ancestral *SulfC* gene early in the *Plutella* lineage, resulting in *GSS1/2* and *GSS3*, and a duplication late during radiation of the genus, which gave rise to *PxGSS1* and *PxGSS2*. *GSS* genes differ in their response to GS, and the encoded enzymes have distinct substrate spectra. *PxGSS1* and *2* are constitutively expressed in the gut of DBM larvae, whereas *PxGSS3* expression is strongly inducible by Trp-GS. *Px*GSS1 has a broad activity range and desulfates all GS classes, *Px*GSS2 targets long-chain Met-GS, and *Px*GSS3 detoxifies Phe- and Trp-GS.

**Fig. 6.**
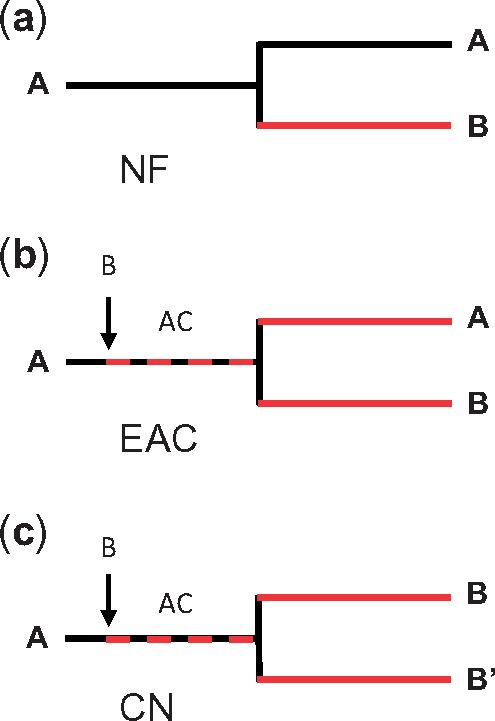
Retention and divergence of duplicate genes under positive selection. Red lines indicate positive, black lines purifying, and dashed red/black lines balancing selection. (*a*) NF. After gene duplication, one copy acquires a novel, adaptive function (B), whereas the other copy retains the ancestral function (A). (*b*) EAC. A novel function (B) emerges before gene duplication, leading to an adaptive conflict (AC) between ancestral function (A) and novel function (B). Gene duplication resolves this conflict. One copy improves the ancestral (A), the other the novel function (B). (*c*) CN. A novel function (B) emerges before gene duplication, leading to an adaptive conflict (AC) between ancestral function (A) and novel function (B). Dosage effects favor duplication and environmental change renders the ancestral function (A) obsolete. Both gene copies are selected for novel functions (B, B′).

As expected, *GSS* genes, which provide the genetic architecture for the central insect counteradaptation against GS-based defenses of their host plants, had evolved under positive selection. Both phylogeny-based and population-based divergence tests congruently found positive selection on both daughter copies of the initial *SulfC* duplication, *GSS1/2* and *GSS3*, and on *PxGSS2* after duplication of *GSS1/2*. These patterns of selection were partly unexpected. The second gene duplication, resulting in *PxGSS1* and *PxGSS2*, displayed a signature typical for NF, with positive selection on *PxGSS2* and purifying selection on *PxGSS1*, but the daughter copies of first gene duplication, *GSS1/2* and *GSS3*, evolved both under positive selection. This is incompatible with NF. Likewise, SF, as an essentially neutral evolutionary process, cannot account for positive selection. By contrast, EAC allows for positive selection on both daughter copies of a duplicated gene, with one copy improving the ancestral and the other copy the novel function. However, both daughter copies of the ancestral *SulfC* gene, *GSS1/2* and *GSS3*, coded supposedly for enzymes with GSS function, whereas the ancestral SulfC likely did not have such an activity. Thus, existing models for gene retention under positive selection do not account for both together, early functional divergence of *GSS* genes and associated nucleotide substitution patterns. Hence, we discovered a new pattern of gene retention, with positive selection for a novel function in both copies of a duplicated gene. We call this new pattern “concerted neofunctionalization” (CN) to contrast it from NF and EAC (fig. 6).

Our initial hypothesis was that different GSS functions had been established stepwise in a coevolutionary arms race, to respond to evolutionary innovations in GS-based plant defense. Therefore, we had expected that GSS3-like activity was first, to detoxify phylogenetically ancient Phe- and Trp-GS, and that GSS1-like activity was second, to enable detoxification of Met-GS, which are an evolutionary innovation in the youngest families of the Brassicales. This scenario expected positive selection during establishment of the initial GSS function (against Phe-/Trp-GS) to have occurred before *SulfC* duplication, as well as purifying selection on *GSS3* and positive selection on *GSS1/2* after *SulfC* duplication. This scenario also implied that ermine moths shifted toward hosts from the order Brassicales before Met GS–producing plants emerged.

Alternately, we could imagine that GSS1-like activity was first and that *GSS3* specialized toward Phe- and Trp-GS after *SulfC* duplication, with a concomitant loss of the enzyme’s activity against Met-GS. Under this scenario, we would expect positive selection during establishment of the initial GSS function (against Phe-/Trp-GS and Met-GS) before *SulfC* duplication and purifying selection on *GSS1/2* to preserve the ability to detoxify Met-GS after *SulfC* duplication. Furthermore, we would expect purifying or relaxed selection on *GSS3* if gene retention after *SulfC* duplication followed the SF model, or positive selection if specialization toward Phe- and Trp-GS were adaptive. This scenario would imply that the insect host shift took place after Met GS–producing plants emerged, making it difficult to find evidence for a coevolutionary arms race.

We found that both daughter copies of the duplicated *SulfC*, *GSS1/2* and *GSS3*, evolved concertedly. This implies that the insect host shift occurred in the presence of host plants that were capable of producing Met-GS, in addition to other classes of GS. Indeed, a recent estimate placed the divergence of the Brassicaceae crown group in the Eocene, at 43.4 Ma (Cardinal-McTeague et al. 2016), predating ermine moth host shift toward GS-containing plants. Thus, the coevolutionary arms race hypothesis does not explain early diversification of *GSS* genes.

Can we instead explain CN of *GSS* genes in the context of the insect host plant shift? We do not know the function of the ancestral *SulfC* gene in ermine moths before host shift. This function existed >20 Ma when the insects fed on unknown host plants; hence, it is virtually impossible to determine the target of the ancestral enzyme. However, absence of GSS activity in other insect arylsulfatases (table 1 and supplementary figs. S4 and S5, Supplementary Material online) suggests that the ancestral SulfC function was not GS related. Thus, it is conceivable that GSS activity emerged during the initial phase of insect host shift, causing an adaptive conflict with the ancestral SulfC function. Antagonistic selective constraints would explain why there is only limited evidence for directional selection prior to *SulfC* gene duplication. In turn, weak evidence for positive selection implies that the novel function, GSS activity, was maladapted for GS detoxification before the insects completed their host shift. Consequently, duplication of *SulfC*, coding for an enzyme with suboptimal GSS activity, should have engendered dosage effects in favor of the new host. This would explain why both daughter copies of *SulfC*, *GSS1/2* and *GSS3*, were retained in the *Plutella* lineage in the first place. Subsequently, both copies diverged under positive selection to counter different features of the plant GS–myrosinase complex, with GSS1/2 detoxifying constitutive levels of all GS classes and GSS3 scavenging surplus Phe- and Trp-GS, which are inducible upon herbivore attack. Hence, we can conclude that the initial *SulfC* duplication was central for the success of the counteradaptation and favored insect host shift toward GS-producing plants.

As discussed above, an adaptive conflict between SulfC and GSS function prior to *SulfC* duplication is plausible. But in contrast to EAC, which predicts that one gene duplicate should have restored the ancestral gene function, that is, SulfC activity, both daughter copies had been selected for a new function, GSS activity. This could indicate that concurrent changes elsewhere in the insect genome compensated the loss of the ancestral SulfC function. Alternately, if this ancestral function had been important for adaptation to the previous but not the new host plants, it would have become dispensable after full transition of the insect to new hosts, and, hence, full transition to the new hosts would have entailed the disappearance of a previously existing adaptive conflict. Thus, CN may be an exceptional outcome of an adaptive conflict, triggered by changes in the environment that render the ancestral function of a duplicate gene pair obsolete. Genome-wide studies suggest that it is not uncommon that both copies of a duplicate gene pair acquire novel expression patterns or that both encoded proteins participate in novel protein–protein interactions (He and Zhang 2005; Assis and Bachtrog 2013). However, it remains unknown whether in any of these cases both copies evolved enzymatic functions that were not encoded by the ancestral gene. Likewise, few empirical studies have distinguished between NF and EAC. Hence, it is unknown how frequently different duplicate gene retention mechanisms contribute to evolutionary innovation. Since environmental change may be a prerequisite for CN, it should be possible to detect other cases of CN in duplicate gene pairs that mediate interactions with the abiotic or biotic environment.

## Materials and Methods

### Chemicals

*Helix pomatia* arylsulfatase, 4-methylumbelliferyl sulfate, 4-methylumbelliferone, and 2-propenyl GS (Sinigrin) were purchased from Sigma Aldrich. In addition, GSs were extracted from seeds of *Eruca sativa* (4-methylthiobutyl GS), *Sinapis alba* (*p*-hydroxybenzyl GS), *Isatis tinctoria* (indol-3-ylmethyl GS), *Brassica oleracea* (4-methylsulfinylbutyl GS), *Sisymbrium officinale* (1-methylethyl GS), *Camelina sativa* (9-methylsulfinylnonyl GS, 10-methylsulfinyldecyl GS) and from leaves of *Brassica napus* (4-pentenyl GS, indol-3ylmethyl GS, 1-methoxyindol-3ylmethyl GS, 4-methoxyindol-3ylmethyl GS) according to a published protocol (Badenes-Perez et al. 2010), and tested as isolated substances or in a mix of several GSs.

### Insect and Plant Cultivation

All strains of *P. xylostella* were continuously mass reared on their standard diet (G88: artificial diet; DBM-C: *Brassica oleracea*; DBM-R: *Brassica napus*; DBM-P: *Pisum sativum*) in an environment-controlled growth chamber adjusted to 16 h light/8 h dark at 21 °C and 58% relative humidity. Plants used for mass rearing of insects were grown in trays (58 × 32 × 11.5 cm) under greenhouse conditions at 21–23 °C, 50–60% relative humidity, and 14 h light/10 h dark cycle, with about 60 plants per tray. Plants for feeding assays were raised under the same conditions, except that plants were separated at the seedling stage and grown individually in single pots. *Arabidopsis thaliana* wildtype and GS mutants were grown with 12 h light/12 h dark at 21 °C and 50–60% relative humidity prior to feeding assays. Assays were carried out by placing *P. xylostella* neonates onto 4-week-old *A. thaliana* wildtype or GS mutants, or onto 5-week-old *Brassica* or *Pisum sativum*, respectively, each with three biological replicates. Rearing was carried out with a photoperiod of 16 h light/8 h dark cycle at 21 °C and 58% relative humidity until larvae reached the fourth instar.

### Real-Time Quantitative PCR

Late fourth instar larvae were separated in guts and rest bodies. RNA was extracted from three pools of each ten guts or rest bodies using the innuPrep DNA/RNA Mini kit (AnalytikJena) according to the manufacturer’s instructions. RNA integrity was verified on a 2100 Bioanalyzer (Agilent). From each pool 1,000 ng of total RNA were reversed transcribed with PrimeScript enzyme (TakaRa) using a 3:1 mix of random and oligo-dT20 primers. Real-time quantitative PCR (qPCR) was performed in optical 96-well plates on a CFX Connect detection system (BioRad), using the Verso SYBR Green 2-Step QRT-PCR Kit (Thermo Scientific) according the manufacturer’s instructions. DNA primers for amplification of genes of interest (GOIs) are listed in supplementary table S5, Supplementary Material online. Full-length amplification of GOI from all *P. xylostella* strains revealed no strain-specific nucleotide polymorphisms in the primer binding sites. Amplification specificity was verified by dissociation curve analysis for each transcript. A standard curve was determined for each primer pair in the CFX Manager (version 3.1) based on Cq values (quantitation cycle) of qPCR running with a dilution series of cDNA pools. The efficiency and amplification factors of each qPCR based on the slope of the standard curve were calculated with the help of the efficiency calculator (www.thermoscientificbio.com/webtools/qpcrefficiency/); last accessed February 24, 2019. The Cq values were determined from two technical replicates of each of the three biological replicates and error bars indicate the standard error of means. *RPS18* (AB180432.1) was used as reference gene. Transcript quantity of GOIs was calculated as RNA molecules of GOI/1000 RNA molecules of *RPS18*.

### NanoLC-HDMS^E^ Analysis of Gel-Separated Proteins

Protein bands of Coomassie Brilliant blue R250 stained gels were cut from the gel matrix and subjected to tryptic digestion (Shevchenko et al. 2007). For nanoUPLC-MS^E^ analysis samples were reconstructed in 50 μl aqueous 1% formic acid. One microliter of the peptide mixture was injected onto an UPLC M-class system (Waters) online coupled to a Synapt G2-si mass spectrometer equipped with a T-WAVE-IMS device (Waters). Samples were first on-line preconcentrated and desalted using a UPLC M-Class Symmetry C18 trap column (100 Å, 180 µm × 20 mm, 5 µm particle size) at a flow rate of 15 µl min^−1^ (0.1% aqueous formic acid). Peptides were eluted onto a ACQUITY UPLC HSS T3 analytical column (100 Å, 75 µm × 200, 1.8 µm particle size) at a flow rate of 350 nl min^−1^ using an increasing acetonitrile gradient from 2% to 90% B over 90 min (Buffers: A, 0.1% formic acid in water; B, 100% acetonitrile in 0.1% formic acid). Eluted peptides were transferred into the mass spectrometer operated in V-mode with a resolving power of at least 20,000 full width at half height). All analyses were performed in a positive electrospray ionization mode. A 100 fmol μl^−1^ human Glu-Fibrinopeptide B in 0.1% formic acid/acetonitrile (1:1 v/v) was infused at a flow rate of 1 μl min^−1^ through the reference sprayer every 45 s to compensate for mass shifts in MS and MS/MS fragmentation mode. During HDMS^E^ analysis, a wave height of 40 V was applied in IMS part of TriWave, and the traveling wave velocity was ramped from 1,000 to 600 m/s. Wave velocities in the trap and transfer cell were set to 311 and 175 m/s and wave heights to 4 and 4 V, respectively. For fragmentation, the collision energy was linearly ramped in the Transfer region of TriWave from 20 to 45 V. The acquisition time in each mode was 0.5 s with a 0.05-s interscan delay. HDMS^E^ data were collected using MassLynx v4.1 software (Waters). Data analysis was performed using ProteinLynx Global Server (PLGS) version 2.5.2 (Waters). The thresholds for low/high energy scan ions and peptide intensity were set at 150, 10, and 750 counts, respectively. The processed data were searched against the *P. xylostella* protein subdatabase constructed from an in-house transcriptome-database by their translation from all six reading frames combined with a database containing common contaminants (human keratins and trypsin). The database searching was performed at a false discovery rate of 2%, with the following search parameters for the minimum numbers of fragments per peptide (1), peptides per protein (1), fragments per protein (4), and maximum number of missed tryptic cleavage sites (1). Searches were restricted to tryptic peptides with a fixed carbamidomethyl modification for Cys residues.

### Antibodies

Polyclonal GSS1/2 and GSS3 rabbit antibodies (Eurogentec, Brussels) were raised against a heterologously expressed and nickel-agarose column purified *Px*GSS1 protein (Xpress System Protein Purification; Invitrogen)^2^ or a *Px*GSS3-specific peptide (LFRDYKPDFEAEGYC; with an extra “C” at the C-terminus to facilitate conjugation), respectively. Preimmune sera as well as immunized sera were tested for specificity and potential background signals using heterologously expressed *PxGSS1*, *2*, and *3*, *PxSulfD*, and *P. xylostella* protein isolated from gut tissue.

### Western Blot Hybridization

Guts from fourth instar larvae were isolated and pools of five guts from the same treatment and strain were kept in phosphate-buffered saline (500 µl) including proteinase inhibitor (cOmplete, Mini, EDTA-free, Roche) on ice. Gut proteins were extracted by vigorous shaking with stainless steel beads for 2 min and subsequent centrifugation at 3,500 × g for 15 min. Protein concentration was determined with a Bradford assay. Abundance of GSS proteins was visualized by Western blotting using a polyclonal GSS1/2 or a GSS3 antibody, followed by hybridization with an horseraddish peroxidase (HRP)-conjugated secondary anti-rabbit antibody and detection with the SuperSignal West Dura Extended Duration Substrate (Pierce). Antibody specificity was verified with heterologously expressed *PxGSS1–3*, and *PxSulfD* loaded onto the same gel. Coomassie blue staining was conducted to verify that approximately the same protein amount was loaded for each gut sample. Gut samples for hybridization with the GSS1/2 antibody were treated with PNGase F (New England BioLabs P0704S) according to the manufacturer’s instructions prior to loading. Similarly, a representative sample for hybridization with the GSS3 antibody was treated with PNGase F prior to loading.

### Heterologous Expression

The cDNAs encoding arylsulfataselike genes of *P*. *xylostella* (*PxGSS1*, *PxGSS2*, *PxGSS3*, and *PxSulfD*), *Bombyx mori SulfC1* (*BmC1*), and *Yponomeuta cagnagella C* (*YcC*) were amplified by PCR using gene-specific primers (supplementary table S1, Supplementary Material online), which included a 5′ Kozak sequence and lacked the native stop codon to facilitate epitope and His-tag fusion expression. PCR products were ligated into a pIB/V5-His TOPO TA vector (Invitrogen), and correct sequence and orientation were verified by Sanger sequencing. *Sf*9 cells were cultivated in GIBCO Sf-900 II SFM (Invitrogen) on six-well plates at 27 °C until 70–90% confluence was achieved. Transfection was performed with FuGENE HD (Promega) following the manufacturer’s protocol. At 72 h after transfection, the culture medium of *Sf*9 cells was harvested and aliquots were directly used for blotting or for activity assays. Expressed proteins were detected with an anti-V5 HRP antibody (Thermo Fisher Scientific) using the SuperSignal West HisProbe Kit (Pierce).

### Arylsulfatase Assays

Quantities of recombinant proteins were adjusted by Western blot analysis of a dilution series, using an anti-V5 HRP antibody. Culture medium containing recombinant arylsulfatases was incubated with a final concentration of 3.5 mM 4-methylumbelliferyl sulfate in the assay buffer (50 mM Tris, 500 mM sodium chloride, 100 µM magnesium chloride, 100 µM manganese chloride, 100 µM calcium chloride, pH 7.5) at 37 °C for 10–180 min. Reactions were stopped by adding four volumes of 0.5 M sodium carbonate buffer (pH 10.5). Ten units of a crude enzyme preparation of *Helix pomatia* containing arylsulfatase activity was used as a positive control, a culture of nontransfected *Sf*9 cells and boiled heterologously expressed insect arylsulfatases were used as negative control and blanks. The formation of 4-methylumbelliferone was measured at 360 nm on an Infinite M200 microplate reader (Tecan) and quantified by comparison with a standard curve obtained from a dilution series of 4-methylumbelliferone.

### GSS Assays

Fifty-microliter-aliquots of recombinant arylsulfatases in 100 mM Tris, pH 7.5, were mixed with 50 µl of 5 mM GS solution or 50 µl of GS extracts for 2 h at ambient temperature. Reactions were stopped with 500 µl methanol and mixtures were centrifuged for 5 min. Two hundred microliters of supernatants were diluted 3-fold with distilled water and subjected to high performance liquid chromatography on an Agilent 1100 HPLC system using a reversed phase C-18 column (Nucleodur Sphinx RP, 250 × 4.6 mm, 5 µm, Macherey-Nagel, Düren, Germany) with a water (A)/acetonitrile (B) gradient (1 min: 1.5% B; 5 min: 1.5–5% B; 2 min: 5–7% B; 10 min: 7–21% B; 5 min, 21–29% B; 0.1 min: 29–100% B; 0.9 min: 100% B; 4 min: 1.5% B; flow rate: 1.0 ml min^−1^). Detection was performed with a photodiode array detector and peaks were integrated at 229 nm. Desulfoglucosinolates were identified based on ultraviolet absorption spectra, retention time, and mass spectra from liquid chromatography-mass spectrometry (LC-MS) analysis conducted with a Bruker Esquire 6000 IonTrap mass spectrometer.

### Molecular Phylogenetic and Evolutionary Analyses

A multiple alignment of arylsulfatase amino acid sequences was conducted with MUSCLE (Edgar 2004) using the web server of the European Bioinformatics Institute (www.ebi.ac.uk/Tools/msa/muscle; last accessed February 24, 2019). All gaps and unreliable positions were removed, resulting in a final alignment of 439 codons. MEGA7 (Kumar et al. 2016) was used for inferring the evolutionary history of sulfatase genes with maximum likelihood based on the General Time Reversible Model (Nei and Kumar 2000). Initial trees for heuristic search were obtained automatically by applying Neighbor-Join and BioNJ algorithms to a matrix of pairwise distances estimated using the Maximum Composite Likelihood approach, and then selecting the topology with superior log likelihood value. A discrete gamma distribution was used to model evolutionary rate differences among sites, with some sites allowed to be evolutionary invariable. The reliability of the tree branching order was tested with 1,000 bootstrap replicates. Codon substitution patterns were analyzed with PAML 4.8 (Yang 2007), using PAMLX (Xu and Yang 2013) as a graphical interface. Analyses were based on the alignment described above and the (unrooted) gene tree obtained with MEGA7. Branch-site tests (Yang and Nielsen 2002; Zhang et al. 2005) were conducted to test for positive selection, using the Holm–Bonferroni procedure (Holm 1979) to correct for multiple testing. Amino acid sites under positive selection were inferred with Bayes empirical Bayes (Yang et al. 2005). Additional tests for positive selection were based on two alternative trees, 1) without sequences from *P. australiana* and 2) with a slightly different branching order, placing *YcC* next to *B. mori* and *S. frugiperda SulfC* sequences instead of grouping it together with *Plutella GSS* genes. These alternative trees yielded essentially the same patterns of and support for positive selection as the tree shown in figure 5. BUSTED (Murrell et al. 2015) and aBSREL (Smith et al. 2015) were carried out under Datamonkey 2.0 (Weaver et al. 2018). McDonald–Kreitman tests for positive selection (McDonald and Kreitman 1991) were conducted online (mkt.uab.es/mkt/; last accessed February 24, 2019; Egea et al. 2008), using distance correction (Jukes and Cantor 1969). For these tests, PAML was used to reconstruct a suite of ancestral sequences for inferring polymorphism under different models, including a neutral model (M0) and branch-site models with *ω*_2_ > 1 (positive selection) or *ω*_2_ = 1 (nearly neutral) for key nodes of the arylsulfatase gene tree. These key nodes were the ancestral *SulfC* gene shared by *Y. cagnagella* and *P. xylostella*, *SulfC* at the time of the first gene duplication in the *Plutella* lineage, *GSS1/2*, and *GSS3* at the time when *P. xylostella* and *P. australiana* lineages separated from a common ancestor.

### *P. australiana* Genome

A female *P. australiana* sample was collected from Cook, Australia (−35.262015, 149.058586) in December 2014. DNA was prepared using phenol-chloroform isolation and the genome sequenced using Illumina HiSeq (Australian Genome Research Facility), generating 33.1 million 100-bp paired end reads. The genome was assembled with ABySS (v1.3.4) using the abyss-pe function, a *k*-mer value of *k* = 64 and minimum base quality *q* = 20 (Kearse et al. 2012). This yielded 1,609,447 scaffolds and N50 of 2,432 bp. Px*GSS1*, *2*, and *3*, and *PxSulfD* nucleotide sequences were BLASTed against the *P. australiana* genome assembly using Geneious (v.10.2) (Simpson et al. 2009) and gene models manually generated based on the exon structure present in *P. xylostella*.

## Supplementary Material

Supplementary Data
