# Supplementary material for: An Insect Counteradaptation against Host Plant Defenses Evolved through Concerted Neofunctionalization
Source: Mol Biol Evol. 2019 Feb 1;36(5):930–41. doi: 10.1093/molbev/msz019 (PMC6501874; doi:10.1093/molbev/msz019)
Supplement: Supplementary Data [file msz019_supp.pdf]

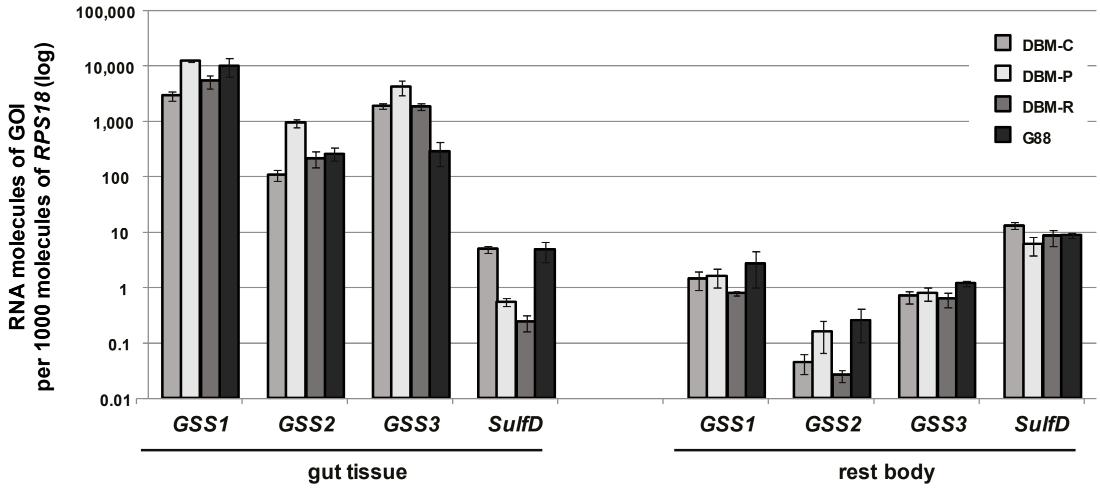

Figure S1. Abundance of *PxGSS1 – 3* and *PxSulfD* transcripts in gut (left) and larval body (right) of four *P. xylostella* strains, DBM-C, -P, -R and G88. Larvae were reared on *Brassica oleracea*, *B. napus*, *Pisum sativum*, or on standard artificial diet, respectively. Shown are results from three biological replicates of ten pooled individuals per strain (± SEM). Genes of interest (GOI) were compared to *RPS18* as a reference gene.

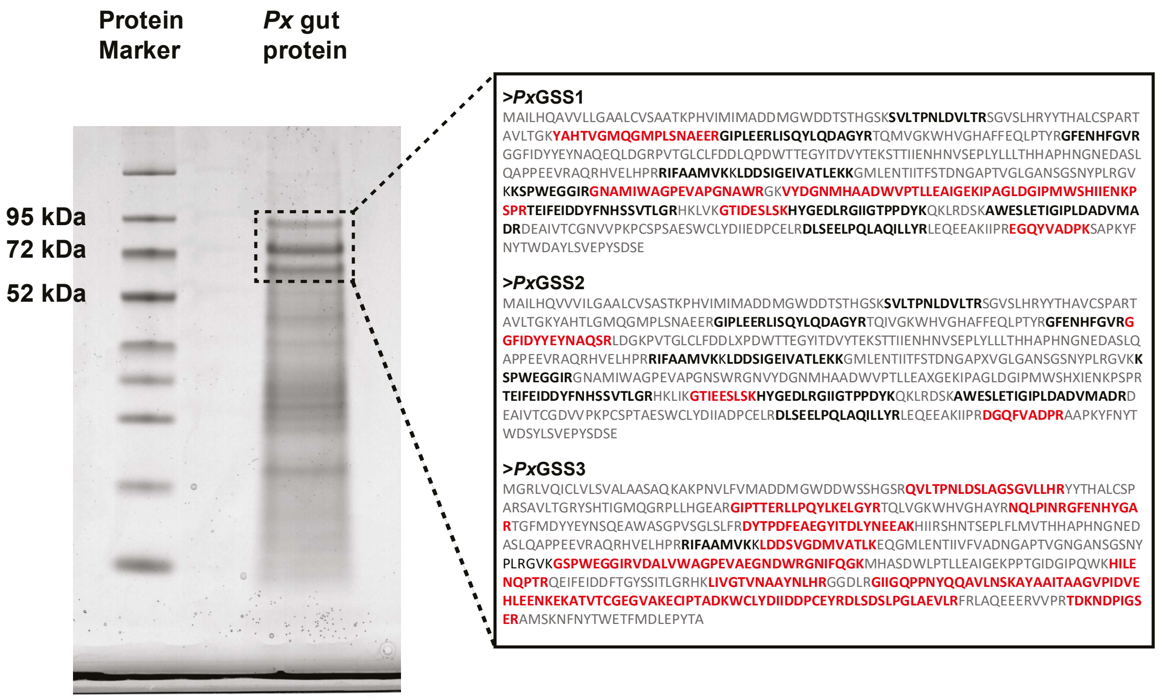

Figure S2. Peptide sequencing by mass spectrometry (MS^E) of DBM-R. Three bands (dotted square region) from an SDS-PAGE protein gel (left) of *P. xylostella* strain DBM-R were subjected to MS^E analysis as described in the methods. The box (right) shows GSS1 – 3 amino acid sequences. Black letters correspond to peptides that are present in more than one GSS, red letters correspond to unique peptides for a given GSS.

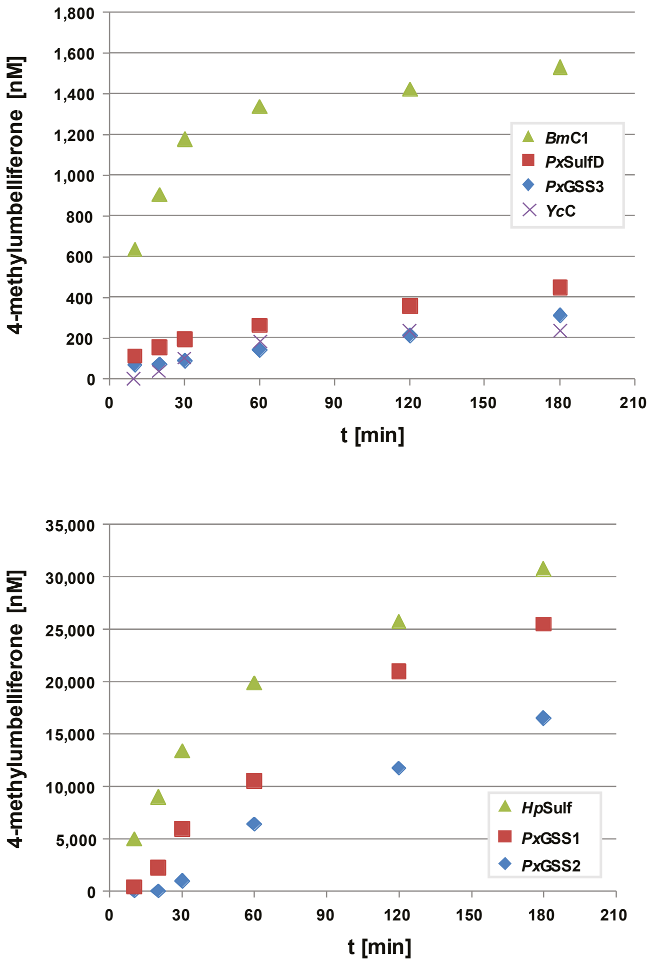

Figure S3. Activity of recombinant arylsulfatases with the arylsulfatase standard substrate 4-methylumbelliferyl sulfate. Heterologously expressed genes from *P. xylostella* (*PxGSS1 – 3* and *PxSulfD*), *B. mori* (*BmC1*) and *Y. cagnagella* (*YcC*) are active sulfatases. *HpSulf*, a sulfatase extracted from the burgundy snail *Helix pomatia* was used as a positive control.

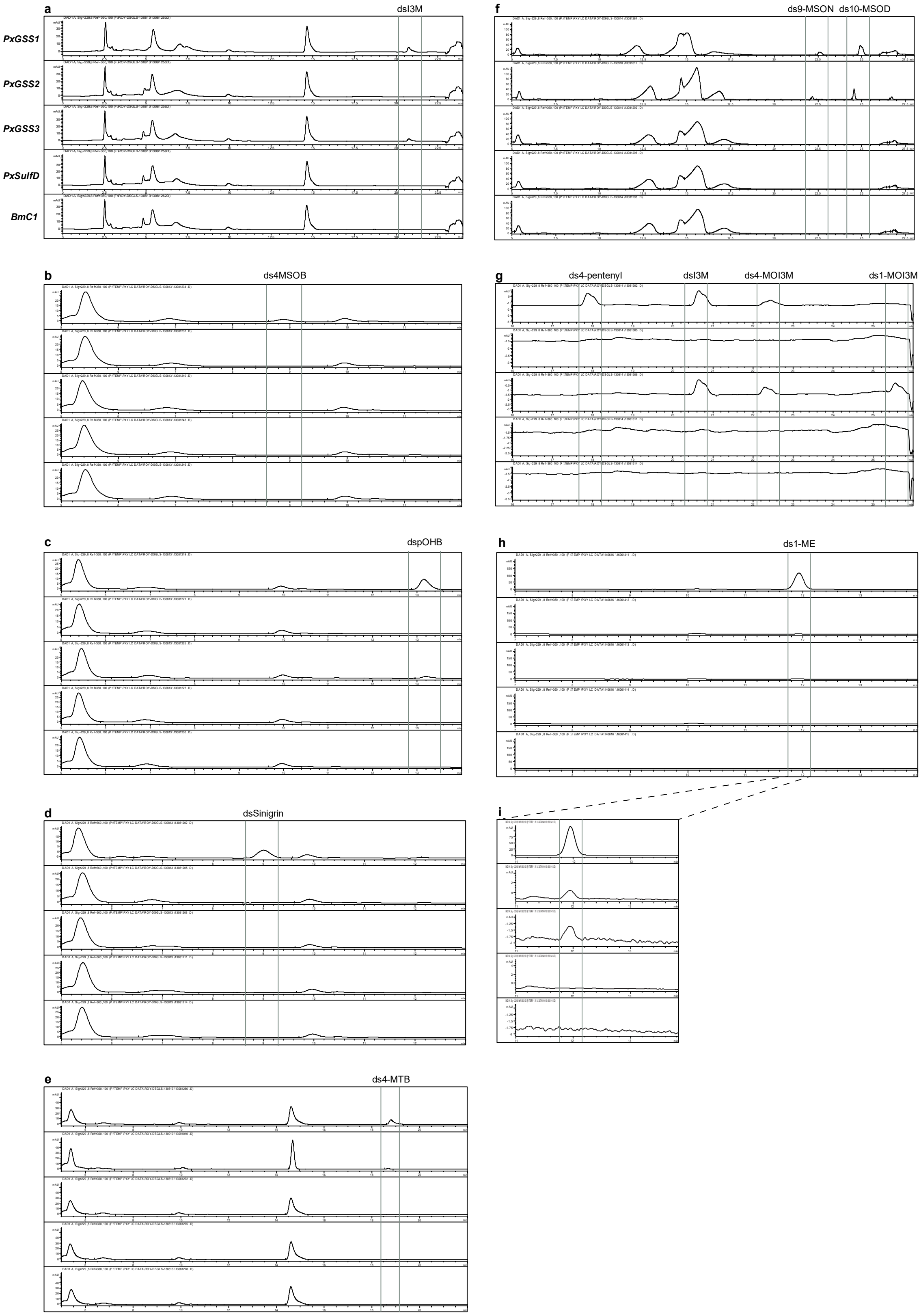

Figure S4. HPLC chromatograms from arylsulfatase enzyme assays. Individual glucosinolates (a-e) or glucosinolate mixtures extracted from *Camelina sativa* (f), *Brassica napus* (g) or *Sisymbrium officinale* (h, i) were used for enzymatic assays with recombinant proteins (*Px*GSS1, *Px*GSS2, *Px*GSS3, *Px*SulfD and *Bm*C1). Desulfo-glucosinolates were identified by HPLC at 229 nm.

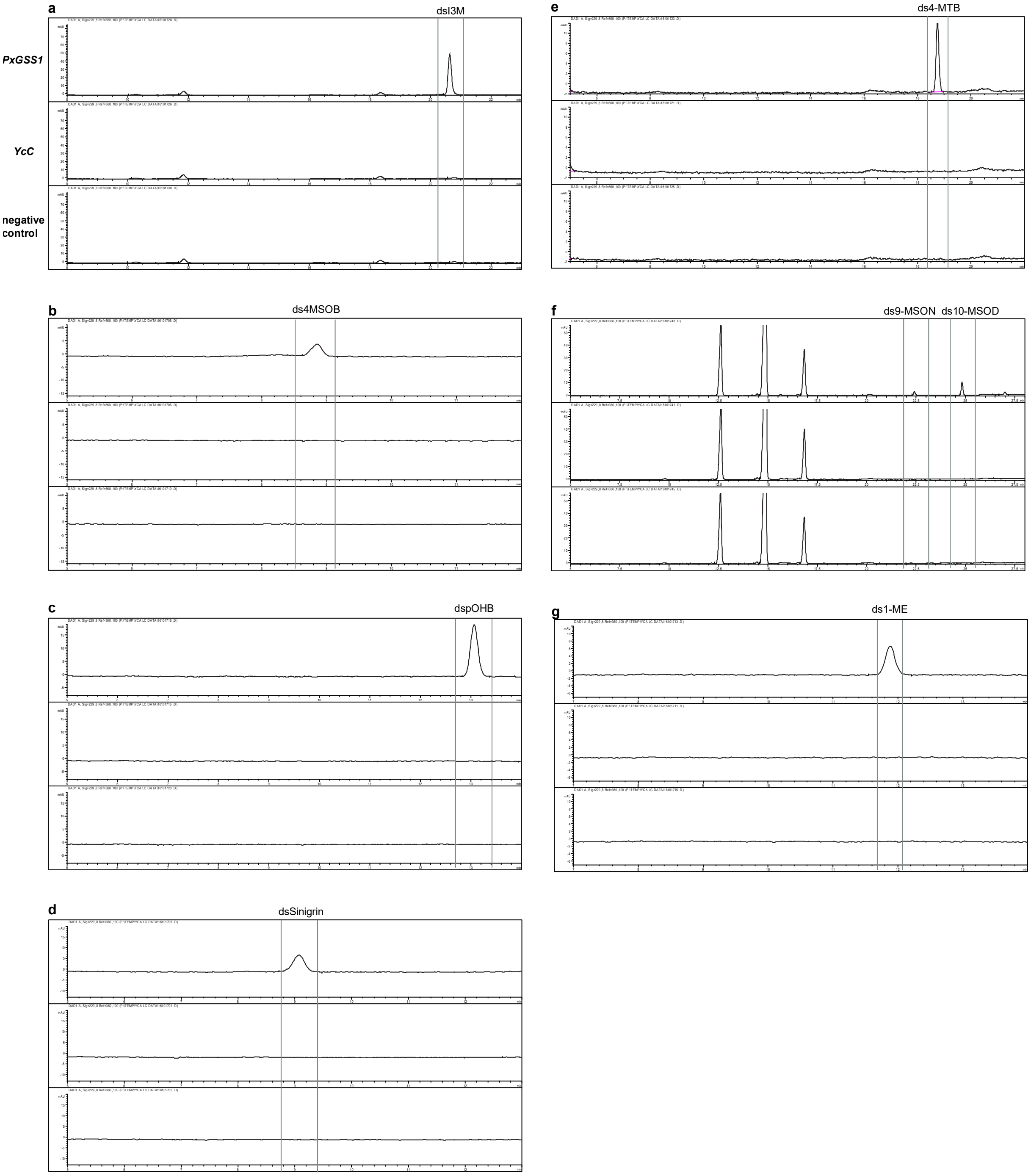

Figure S5. HPLC chromatograms from arylsulfatase enzyme assays (continued). Individual glucosinolates (a-e) or glucosinolate mixtures extracted from *Camelina sativa* (f) or *Sisymbrium officinale* (g) were used for enzymatic assays with recombinant proteins (*Px*GSS1, *Yc*C). Desulfo-glucosinolates were identified by HPLC at 229 nm.

Table S1. Results from branch-site tests for positive selection – Support for positive selection along branches.

Foreground branch in Figure 5	lnL(ω_2 = 1.0)	lnL(ω_2 ≥ 1.0)	2Δ*l*	*p* (*df*=1)	adjusted (*df*=1)
(((((((PxGSS1a, PxGSS1b) #1, (PxGSS2a, PxGSS2b) #2) #3, PaGSS1/2 #4) #5, ((PxGSS3a, PxGSS3b) #6, PaGSS3 #7) #8) #9, YcC #10) #11, ((BmC1 #12, BmC2 #13) #14, (SfC1 #15, SfC2 #16) #17) #18), ((((PxD, PaD), YcD), (BmD, SfD)), ((PxB, YcB), (BmB, SfB))), (BmA, SfA));					
#1	-21356.4	-21356.4	0.0	0.979	n.s.
#2	-21354.1	-21350.0	8.2	0.004	≤ 0.05
#3	-21355.7	-21353.5	4.3	0.038	n.s.
#4	-21353.8	-21343.7	20.2	0.000	0.000
#5	-21328.2	-21311.6	33.1	0.000	0.000
#6	-21356.4	-21354.9	3.0	0.085	n.s.
#7	-21356.4	-21356.4	0.0	1.000	n.s.
#8	-21339.6	-21322.3	34.7	0.000	0.000
#9	-21350.3	-21347.0	6.7	0.010	n.s.
#10	-21347.4	-21342.2	10.4	0.001	≤ 0.05
#11	-21356.4	-21355.8	1.2	0.269	n.s.
#12	-21354.9	-21353.6	2.5	0.113	n.s.
#13	-21356.4	-21351.8	9.1	0.003	≤ 0.05
#14	-21344.5	-21340.5	8.0	0.005	≤ 0.05
#15	-21349.1	-21348.7	0.7	0.389	n.s.
#16	-21343.9	-21340.5	6.9	0.009	n.s.
#17	-21352.5	-21348.2	8.6	0.003	≤ 0.05
#18	-21352.3	-21346.5	11.7	0.001	≤ 0.01

Table S2. Results from branch-site tests for positive selection – Site classes.

Foreground branch in Figure 5	site class	0	1	2a	2b
	proportion	0.86	0.13	0.01	0.00
#2	background ω	0.10	1	0.10	1
	foreground ω	0.10	1	98	98
	proportion	0.86	0.12	0.02	0.00
#4	background ω	0.10	1	0.10	1
	foreground ω	0.10	1	85	85
	proportion	0.76	0.10	0.12	0.02
#5	background ω	0.09	1	0.09	1
	foreground ω	0.09	1	56	56
	proportion	0.78	0.10	0.10	0.01
#8	background ω	0.09	1	0.09	1
	foreground ω	0.09	1	999	999
	proportion	0.80	0.11	0.07	0.01
#10	background ω	0.09	1	0.09	1
	foreground ω	0.09	1	365	365

Table S3. Results from branch-site tests for positive selection – Positively selected sites.

																													1	2		3	4	5	6	7	8	9	10	11	12	13	14	15	16	17	18	19	20	21	22	23	24	25	26	27	28	29	30
	1	2	3	4	5	6	7	8	9	10	11	12	13	14	15	16	17	18	19	20	21	22	23	24	25	26	27	28	29	30	31	32	33	34	35	36	37	38	39	40	41	42	43	44	45	46	47	48	49	50	51	52	53	54	55	56	57	58	59	60
Px GSS1	-	-	-	-	-	-	M	A	I	L	H	Q	A	V	V	L	L	G	A	A	L	C	-	-	V	S	A	A	T	K	P	-	H	V	**I**	**M**	I	M	A	D	D	M	G	W	D	D	**T**	S	T	H	G	S	**K**	**S**	V	L	T	P	N	L
Px GSS2	-	-	-	-	-	-	M	A	I	L	H	Q	V	V	V	I	L	G	A	A	L	C	-	-	V	S	A	S	T	K	P	-	H	V	**I**	**M**	I	M	A	D	D	M	G	W	D	D	**T**	S	T	H	G	S	**K**	**S**	V	L	T	P	N	L
Pa GSS1/2	-	-	-	-	-	-	M	A	I	L	H	Q	A	V	V	L	L	G	A	A	L	C	-	-	V	S	A	A	T	K	P	-	H	V	**I**	**M**	I	M	A	D	D	M	G	W	D	D	**T**	S	T	H	G	S	**K**	**S**	V	L	T	P	N	L
Px GSS3	-	-	-	-	-	-	M	G	R	L	V	Q	I	C	L	V	L	S	V	A	L	A	A	-	S	A	Q	K	A	K	P	-	N	V	**L**	F	**V**	M	A	D	D	M	G	W	D	D	**W**	S	S	H	G	S	R	Q	V	L	T	P	N	L
Pa GSS3	-	-	-	-	-	-	M	G	R	L	V	Q	I	C	L	V	L	S	V	A	L	A	A	-	S	A	Q	K	A	K	P	-	N	V	**L**	F	**V**	M	A	D	D	M	G	W	D	D	**W**	S	S	H	G	S	R	Q	V	L	T	P	N	L
Yc C	-	-	-	-	-	-	M	F	L	L	A	A	F	S	A	F	L	C	Y	A	F	T	-	-	R	S	E	V	T	R	P	-	N	V	M	L	L	I	A	D	D	M	G	W	N	D	V	S	F	H	G	S	R	Q	I	P	T	P	N	I
Bm C1	-	-	-	-	-	-	M	F	V	F	R	V	L	L	I	S	V	L	M	L	V	A	E	-	S	T	K	R	K	S	S	-	N	I	V	L	I	V	A	D	D	L	G	W	N	D	I	S	F	H	G	S	N	Q	I	L	T	P	N	I
Bm C2	-	-	-	-	-	-	-	M	Y	N	T	I	L	F	L	I	L	N	W	I	V	N	A	-	S	T	Q	Q	K	S	P	-	N	I	V	L	I	I	A	D	D	L	G	W	D	D	V	S	F	H	G	S	D	Q	I	L	T	P	N	I
Sf C1	-	-	-	-	-	-	M	G	I	L	S	V	L	P	V	L	I	F	T	G	F	I	A	S	Y	C	D	A	S	K	P	-	N	I	V	F	I	L	A	D	D	L	G	W	N	D	V	S	F	H	G	S	D	Q	I	L	T	P	N	I
Sf C2	-	-	-	-	-	-	-	-	-	-	M	L	S	L	V	V	S	I	A	I	V	T	L	-	V	C	K	A	D	K	P	-	N	I	V	F	I	I	A	D	D	L	G	W	D	D	V	S	F	H	G	S	D	Q	I	L	T	P	N	I
Px D	-	-	-	-	-	M	L	Y	K	W	A	R	W	F	P	A	V	V	W	L	A	-	-	-	T	V	A	A	Q	Q	P	-	H	V	V	F	I	M	V	D	D	M	G	W	N	D	V	S	F	H	G	S	D	Q	I	Q	T	P	N	I
Pa D	-	-	-	-	-	M	L	Y	K	W	A	R	W	F	P	V	V	V	W	L	A	-	-	-	A	V	A	A	Q	Q	P	-	H	I	V	L	I	M	V	D	D	M	G	W	N	D	V	S	Y	H	G	S	D	Q	I	Q	T	P	N	I
Yc D	-	-	-	-	-	M	A	L	T	W	Q	R	C	C	A	A	L	A	L	M	T	V	F	-	A	V	S	Q	K	P	P	-	H	V	V	F	I	M	V	D	D	M	G	W	N	D	V	S	F	H	G	S	D	Q	I	Q	T	P	N	I
Bm D	-	-	M	H	K	L	N	A	I	W	S	L	C	W	A	L	F	V	I	A	S	A	L	-	A	Y	P	R	P	N	S	-	N	V	V	F	I	M	V	D	D	M	G	W	N	D	V	S	F	H	G	S	D	Q	I	L	T	P	N	I
Sf D	M	C	N	V	N	Q	Y	V	S	W	K	Q	W	W	I	I	L	A	M	I	S	T	T	I	S	G	Y	R	Q	Q	P	S	N	I	V	F	I	M	V	D	D	M	G	W	N	D	V	S	F	H	G	S	D	Q	I	L	T	P	N	I
Px B	-	-	-	-	-	-	-	-	-	-	-	-	-	-	-	-	-	-	-	-	-	-	-	-	-	V	K	Q	Q	P	P	-	N	I	V	I	I	M	A	D	D	L	G	W	N	D	V	S	F	H	G	S	N	Q	I	L	T	P	N	I
Yc B	-	-	-	-	-	-	-	M	D	P	Q	L	I	G	F	V	L	V	A	T	S	V	L	-	S	A	W	A	G	P	P	-	N	I	V	V	I	M	A	D	D	M	G	W	N	D	V	S	Y	H	G	S	D	Q	I	L	T	P	N	I
Bm B	-	-	-	-	-	-	M	F	F	T	F	L	F	I	I	G	T	L	I	L	N	T	L	-	S	E	D	I	V	K	P	-	N	I	V	F	I	V	A	D	D	L	G	W	D	D	V	S	I	H	G	S	D	Q	I	M	T	P	N	I
Sf B	-	-	-	-	-	-	-	-	M	R	L	I	F	Y	V	L	S	L	V	V	L	I	K	-	C	E	S	L	S	K	P	-	N	I	V	F	I	V	A	D	D	L	G	W	S	D	V	S	F	H	G	S	D	Q	I	M	T	P	N	I
Bm A	-	-	-	-	-	-	-	M	S	W	L	V	L	I	V	I	L	G	-	-	-	-	-	-	V	A	S	A	T	R	P	-	N	I	I	F	F	L	A	D	D	L	G	W	N	D	V	G	F	H	G	S	N	Q	I	P	T	P	N	L
Sf A	-	-	-	-	-	-	-	-	-	-	-	M	L	L	L	T	L	W	S	V	L	C	V	-	A	S	A	Q	V	R	P	-	N	I	V	I	F	L	A	D	D	L	G	W	N	D	V	G	F	H	G	S	N	Q	I	P	T	P	N	I
																														.		:	:	:	.	.	:	.	*	*	:	*	*	.	*		.		*	*			.	:		*	*	*	:

	31	32	33	34	35	36	37	38	39	40	41	42	43	44	45	46	47	48	49	50	51	52	53	54	55	56	57	58	59	60	61	62	63	64	65	66	67	68	69	70		71	72	73	74	75	76	77	78	79	80	81	82	83	84	85	86	87	88	89
	61	62	63	64	65	66	67	68	69	70	71	72	73	74	75	76	77	78	79	80	81	82	83	84	85	86	87	88	89	90	91	92	93	94	95	96	97	98	99	100	101	102	103	104	105	106	107	108	109	110	111	112	113	114	115	116	117	118	119	120
Px GSS1	D	V	L	T	R	S	G	V	S	L	H	R	Y	Y	T	H	A	L	C	S	P	A	R	T	A	V	L	T	G	K	Y	A	H	T	V	G	M	Q	G	M	-	P	L	S	N	**A**	E	E	R	G	I	P	L	E	E	R	L	**I**	S	Q
Px GSS2	D	V	L	T	R	S	G	V	S	L	H	R	Y	Y	T	H	A	V	C	S	P	A	R	T	A	V	L	T	G	K	Y	A	H	T	L	G	M	Q	G	M	-	P	L	S	N	**A**	E	E	R	G	I	P	L	E	E	R	L	**I**	S	Q
Pa GSS1/2	D	V	L	T	R	S	G	V	S	L	H	R	Y	Y	T	H	A	L	C	S	P	A	R	T	A	V	L	T	G	K	Y	A	H	T	V	G	M	Q	G	M	-	P	L	S	N	**A**	E	E	R	G	I	P	L	E	E	R	L	**I**	S	Q
Px GSS3	D	S	L	A	G	S	G	V	L	L	H	R	Y	Y	T	H	A	L	C	S	P	A	R	S	A	V	L	T	G	**R**	Y	**S**	H	T	I	G	M	Q	G	R	-	P	L	L	**H**	G	E	**A**	R	G	I	P	T	T	E	R	L	L	P	Q
Pa GSS3	D	S	L	A	G	S	G	V	L	L	H	R	Y	Y	T	H	A	L	C	S	P	A	R	S	A	V	L	T	G	**R**	Y	**S**	H	T	I	G	M	Q	G	R	-	P	L	L	**H**	G	E	**A**	R	G	I	P	Q	S	E	R	L	L	P	Q
Yc C	D	M	L	A	Y	S	G	V	A	L	Q	R	Y	Y	S	H	A	Q	C	T	P	S	R	S	A	I	L	T	G	K	F	A	H	K	I	G	M	Q	G	Y	-	P	L	T	N	S	E	D	R	G	L	P	L	T	E	K	L	F	P	E
Bm C1	D	L	L	S	L	S	G	K	G	L	G	R	Y	Y	S	H	C	I	C	T	P	S	R	S	A	L	M	T	G	K	Y	A	H	R	I	G	M	Q	G	F	-	P	L	S	N	S	E	D	R	G	L	P	T	N	E	K	I	L	P	Q
Bm C2	D	L	L	A	Y	S	G	K	A	L	G	R	Y	Y	T	H	C	I	C	T	P	S	R	A	A	L	L	T	G	K	Y	A	H	R	I	G	M	Q	G	Y	-	P	L	T	N	S	E	D	R	G	L	P	T	T	E	K	I	L	P	Q
Sf C1	D	S	L	A	Y	S	G	V	A	F	G	R	Y	Y	S	H	A	I	C	T	P	S	R	A	A	L	L	T	G	K	Y	S	Y	K	T	G	M	Q	G	N	-	P	L	G	M	S	E	D	R	S	I	P	T	N	V	R	L	L	P	Q
Sf C2	D	L	L	A	Y	S	G	V	T	L	G	R	Y	Y	S	H	A	I	C	T	P	S	R	S	A	L	L	T	G	K	Y	S	H	K	I	G	M	Q	G	Y	P	P	L	T	M	S	D	D	R	G	I	P	T	D	L	K	L	L	P	Q
Px D	D	T	L	A	Y	K	G	V	I	L	Q	Q	Y	Y	S	E	A	I	C	T	P	A	R	T	A	L	L	T	G	K	Y	P	M	R	L	G	M	H	G	F	-	P	L	Y	N	S	E	D	R	G	I	P	L	T	E	R	L	L	P	S
Pa D	D	T	L	A	Y	K	G	V	I	L	Q	Q	Y	Y	S	E	A	I	C	T	P	A	R	T	A	L	L	T	G	K	Y	P	M	R	L	G	M	H	G	F	-	P	L	Y	N	S	E	D	R	G	I	P	L	T	E	R	L	L	P	S
Yc D	D	T	L	A	Y	Q	G	V	I	L	Q	Q	Y	Y	S	E	A	M	C	T	P	A	R	T	A	L	L	T	G	K	Y	P	M	R	L	G	M	H	G	F	-	P	L	F	N	S	E	D	R	G	I	P	L	T	E	R	L	L	P	S
Bm D	D	T	L	A	Y	Q	G	V	I	L	Q	Q	Y	Y	S	E	A	I	C	T	P	A	R	T	A	L	L	T	G	K	Y	P	M	R	L	G	M	H	G	S	-	P	L	F	N	S	E	D	R	G	I	P	L	T	E	R	L	L	P	S
Sf D	D	S	L	A	Y	Q	S	A	I	L	H	H	Y	Y	S	E	A	I	C	T	P	A	R	T	A	L	L	T	G	K	Y	P	M	R	L	G	M	H	G	L	-	P	L	F	N	S	E	D	R	G	I	P	L	T	E	R	L	L	P	S
Px B	D	A	I	M	Y	T	G	V	S	L	Q	Q	Y	Y	T	D	A	W	G	S	A	A	R	T	A	F	L	T	G	K	Y	P	M	R	L	G	M	Q	G	P	-	S	I	M	A	H	E	D	R	G	L	P	S	E	E	T	T	L	P	Q
Yc B	D	V	I	G	Y	Q	G	V	M	L	H	Q	Y	Y	T	D	A	F	G	T	P	S	R	S	A	F	F	T	G	K	Y	P	S	T	T	G	T	Q	S	E	-	S	I	R	A	A	E	D	R	G	L	S	V	T	E	K	L	L	P	S
Bm B	D	I	M	G	Y	E	G	T	I	L	H	Q	Y	Y	T	D	S	L	G	T	A	S	R	S	A	L	F	T	G	K	Y	A	I	R	L	G	T	H	G	S	-	S	I	A	A	T	E	D	G	G	V	P	L	S	E	R	L	L	P	S
Sf B	D	V	L	A	Y	Q	G	I	I	I	P	Q	Y	Y	T	D	T	R	G	T	P	A	R	S	A	L	F	T	G	K	Y	P	L	R	M	G	T	Q	G	I	-	S	I	A	S	A	E	D	R	G	L	P	T	T	E	A	L	L	P	Q
Bm A	D	S	L	G	V	S	G	L	M	L	H	N	Y	Y	T	S	P	I	C	S	P	S	R	A	A	L	M	T	G	K	Y	P	I	H	T	G	M	Q	H	G	-	V	I	Y	G	A	E	P	R	G	L	P	L	N	E	K	I	L	P	Q
Sf A	D	A	L	A	Y	S	G	L	I	L	Q	N	Y	Y	V	T	P	I	C	T	P	S	R	A	A	L	M	T	G	K	Y	P	I	H	T	G	M	Q	H	T	-	V	I	R	G	E	E	P	R	G	L	P	L	S	E	K	I	L	P	Q
	*		:				.			:		.	*	*						:	.	:	*	:	*	.	:	*	*	.	.	.				*		:					:				:			.	:	.						:	.	.

	90	91	92	93	94	95	96	97	98	99	100	101	102	103	104	105	106	107	108	109	110	111	112	113	114	115	116	117	118	119	120	121	122	123	124	125	126	127	128	129	130	131	132	133	134	135	136	137	138	139	140			141	142					
	121	122	123	124	125	126	127	128	129	130	131	132	133	134	135	136	137	138	139	140	141	142	143	144	145	146	147	148	149	150	151	152	153	154	155	156	157	158	159	160	161	162	163	164	165	166	167	168	169	170	171	172	173	174	175	176	177	178	179	180
Px GSS1	Y	L	Q	D	**A**	G	Y	R	T	Q	M	V	G	K	W	H	V	G	H	A	F	F	E	Q	L	P	T	Y	R	G	F	E	N	H	F	G	V	R	G	G	F	I	D	Y	Y	E	Y	N	A	Q	E	-	-	Q	**L**	-	D	G	R	P
Px GSS2	Y	L	Q	D	**A**	G	Y	R	T	Q	I	V	G	K	W	H	V	G	H	A	F	F	E	Q	L	P	T	Y	R	G	F	E	N	H	F	G	V	R	G	G	F	I	D	Y	Y	E	Y	N	A	Q	S	-	-	R	**L**	-	D	G	K	P
Pa GSS1/2	Y	L	Q	D	**A**	G	Y	R	T	Q	I	V	G	K	W	H	V	G	H	A	F	F	E	Q	L	P	T	Y	R	G	F	E	N	H	F	G	V	R	G	G	F	I	D	Y	Y	E	Y	N	A	Q	E	-	-	Q	**L**	-	D	G	R	P
Px GSS3	Y	L	K	**E**	L	G	Y	R	T	Q	L	V	G	K	W	H	V	G	H	A	Y	R	**N**	Q	L	P	**I**	N	R	G	F	E	N	H	Y	G	A	R	**T**	G	F	**M**	D	Y	Y	E	Y	N	S	Q	E	-	-	A	W	-	A	S	G	P
Pa GSS3	Y	L	K	**E**	L	G	Y	R	T	Q	L	V	G	K	W	H	V	G	H	A	Y	R	**N**	Q	L	P	**I**	N	R	G	F	E	N	H	Y	G	A	R	**T**	G	F	**M**	D	Y	Y	E	Y	N	S	Q	E	-	-	A	W	-	A	S	G	P
Yc C	Y	M	K	D	L	G	Y	A	T	H	L	V	G	K	W	H	V	G	R	S	R	Y	S	Y	L	P	T	F	R	G	F	D	T	H	Y	G	H	H	G	G	Y	I	D	Y	Y	E	Y	M	D	E	E	-	-	N	W	-	T	S	G	K
Bm C1	Y	L	K	E	L	G	Y	S	T	H	L	V	G	K	W	H	I	G	E	S	R	T	E	F	L	P	T	E	R	G	F	D	S	H	F	G	H	R	G	G	F	L	D	Y	Y	E	Y	T	L	D	E	-	-	K	W	-	A	T	G	T
Bm C2	Y	L	K	E	L	G	Y	S	T	H	L	V	G	K	W	H	V	G	Q	S	R	T	E	Y	L	P	M	Q	R	G	F	D	S	H	F	G	H	R	G	G	Y	V	D	Y	Y	E	Y	T	L	D	E	-	-	T	W	-	S	T	G	E
Sf C1	Y	F	K	D	V	G	Y	S	T	H	L	V	G	K	W	H	A	G	S	S	R	R	E	Y	L	P	T	S	R	G	F	D	T	Y	I	G	H	R	G	G	Y	I	D	Y	Y	E	Y	T	V	T	E	-	-	V	W	-	E	D	G	P
Sf C2	Y	F	K	D	V	G	Y	S	T	Q	L	I	G	K	W	H	V	G	A	S	R	E	E	Y	L	P	T	N	R	G	F	D	S	H	F	G	H	R	G	G	Y	L	D	Y	Y	E	Y	T	Y	E	E	-	-	L	W	-	S	V	G	H
Px D	Y	L	K	E	L	G	Y	A	T	H	L	V	G	K	W	H	V	G	M	S	R	D	E	F	L	P	T	N	R	G	Y	D	T	H	Y	G	M	R	G	G	Y	V	D	Y	Y	T	Y	N	K	I	E	-	-	T	W	P	D	G	R	T
Pa D	Y	L	K	D	L	G	Y	A	T	H	L	V	G	K	W	H	V	G	M	S	R	D	E	F	L	P	T	N	R	G	Y	D	T	H	Y	G	M	R	G	G	Y	V	D	Y	Y	T	Y	N	K	I	E	-	-	T	W	P	D	G	R	T
Yc D	Y	L	K	D	L	G	Y	S	T	H	L	V	G	K	W	H	V	G	M	S	R	D	E	Y	L	P	T	N	R	G	Y	D	S	H	Y	G	M	R	G	G	F	V	D	Y	Y	T	Y	N	K	I	E	-	-	S	W	P	D	G	R	T
Bm D	Y	L	K	E	L	G	Y	S	T	H	L	V	G	K	W	H	V	G	M	S	R	E	E	F	L	P	T	S	R	G	Y	E	S	H	Y	G	M	R	G	G	F	I	D	Y	Y	T	Y	T	N	I	E	-	-	M	W	P	S	G	R	Q
Sf D	Y	L	K	E	L	G	Y	A	T	H	L	V	G	K	W	H	V	G	M	S	R	P	E	Y	L	P	T	S	R	G	Y	D	H	H	Y	G	M	R	G	G	Y	I	D	Y	Y	T	Y	N	K	V	E	-	-	T	W	P	N	G	R	L
Px B	R	L	Q	Q	L	G	Y	A	T	H	L	V	G	K	W	G	L	G	K	S	R	P	A	S	L	P	T	N	R	G	Y	D	S	F	F	G	F	L	D	D	S	I	D	Y	Y	T	F	D	H	I	E	-	-	N	Y	-	N	N	S	Q
Yc B	Y	L	K	D	L	G	Y	S	T	H	L	V	G	K	W	G	L	G	K	S	R	E	Q	Y	L	P	T	N	R	G	F	D	S	F	F	G	F	L	D	G	A	V	D	Y	Y	T	Y	N	L	I	E	-	-	K	W	-	N	R	T	E
Bm B	Y	L	Q	D	A	G	Y	T	T	H	L	L	G	K	W	Q	L	G	H	S	R	K	S	Y	L	P	T	S	R	G	F	D	T	F	Y	G	F	V	T	G	S	V	D	Y	Y	T	Y	S	H	V	E	-	-	S	F	-	N	G	S	A
Sf B	Y	L	Q	E	L	G	Y	S	T	H	L	I	G	K	W	G	L	G	K	S	R	E	H	Y	L	P	T	N	R	G	F	D	T	F	Y	G	F	T	G	S	S	V	D	Y	Y	T	Y	N	H	V	E	-	-	T	W	-	N	D	T	V
Bm A	Y	L	K	D	L	G	Y	K	T	H	L	V	G	K	W	H	L	G	S	Y	K	K	E	Y	L	P	L	N	R	G	F	D	S	H	V	G	F	W	T	G	R	I	D	M	Y	D	H	T	T	M	E	Q	G	S	W	-	-	-	-	-
Sf A	Y	L	K	D	L	G	Y	K	T	H	L	V	G	K	W	H	L	G	H	Y	K	R	E	Y	L	P	M	S	R	G	F	D	T	H	L	G	F	W	T	G	K	I	D	L	Y	D	H	T	N	M	E	I	G	T	W	-	-	-	-	-
		:	:	:		*	*		*	:	:	:	*	*	*			*							*	*			*	*	:	:		.		*				.		:	*		:		.				.									

			143	144	145	146	147	148	149	150	151	152	153	154	155	156	157	158		159	160	161	162	163	164	165	166	167	168	169	170	171	172	173	174	175	176	177	178	179	180	181	182	183	184	185	186	187	188	189	190	191	192	193	194	195	196	197	198	
	181	182	183	184	185	186	187	188	189	190	191	192	193	194	195	196	197	198	199	200	201	202	203	204	205	206	207	208	209	210	211	212	213	214	215	216	217	218	219	220	221	222	223	224	225	226	227	228	229	230	231	232	233	234	235	236	237	238	239	240
Px GSS1	V	T	G	L	C	L	F	**D**	D	L	Q	P	D	W	**T**	T	E	G	-	Y	I	T	D	V	Y	T	E	K	S	T	T	I	I	E	N	H	N	V	S	E	P	L	Y	L	L	L	T	H	H	A	P	H	N	G	N	E	D	A	S	L
Px GSS2	V	T	G	L	C	L	F	**D**	D	L	K	P	D	W	**T**	T	E	G	-	Y	I	T	D	V	Y	T	E	K	S	T	T	I	I	E	N	H	N	V	S	E	P	L	Y	L	L	L	T	H	H	A	P	H	N	D	N	E	D	A	S	L
Pa GSS1/2	V	T	G	L	C	L	F	**D**	D	L	Q	P	D	W	**T**	T	E	G	-	Y	I	T	D	V	Y	T	E	K	S	**V**	N	I	I	E	N	H	N	V	S	E	P	L	Y	L	L	L	T	H	H	A	P	H	N	G	N	E	D	A	S	L
Px GSS3	V	S	G	L	S	L	F	R	D	Y	T	P	D	F	**E**	A	E	G	-	Y	I	T	D	L	Y	**N**	E	E	A	**K**	H	I	I	R	S	H	N	T	S	E	P	L	F	L	M	V	T	H	H	A	P	H	N	G	N	E	D	A	S	L
Pa GSS3	V	S	G	L	S	L	F	R	D	Y	K	P	D	F	**E**	A	E	G	-	Y	I	T	D	L	Y	**N**	E	E	A	**K**	H	I	I	R	S	H	N	T	S	E	P	L	F	L	M	V	T	H	H	A	P	H	N	G	N	E	D	A	S	L
Yc C	V	S	G	L	D	L	Y	R	N	V	T	A	D	W	D	A	E	G	-	Y	V	T	D	L	Y	N	A	E	A	V	K	I	I	G	N	H	D	T	S	K	P	L	F	L	T	V	A	H	N	A	P	H	C	A	L	T	G	A	I	L
Bm C1	V	T	G	Y	G	L	F	K	N	K	T	A	A	W	G	V	E	G	-	Y	L	T	D	V	Y	T	E	E	A	K	K	V	I	K	N	H	D	K	K	K	P	L	F	L	M	L	A	H	N	A	P	H	S	A	N	E	P	A	I	V
Bm C2	V	T	G	F	G	L	F	R	N	E	T	P	A	W	D	V	E	G	-	Y	L	T	D	V	Y	T	E	E	A	K	S	V	I	K	L	H	D	V	N	K	P	L	F	L	M	L	A	H	N	A	P	H	A	A	N	Y	P	S	I	V
Sf C1	V	S	G	L	A	L	F	R	N	L	S	A	A	W	D	V	E	G	-	Y	I	T	D	V	Y	T	D	E	A	V	K	V	I	D	D	H	D	S	S	N	P	L	F	L	M	V	T	H	N	A	P	H	A	G	G	D	T	V	V	L
Sf C2	V	S	G	L	T	L	F	R	N	L	T	P	A	W	D	V	E	G	-	Y	I	T	D	V	Y	T	D	H	A	V	S	V	I	K	N	H	D	T	S	S	P	L	F	L	M	V	A	H	N	A	P	H	S	A	N	D	G	A	P	L
Px D	M	F	G	L	D	L	F	D	G	F	K	A	Q	D	K	E	Q	R	-	Y	L	G	D	A	L	T	D	R	A	I	N	I	I	R	R	H	N	A	S	Q	P	L	F	L	H	L	A	H	N	A	P	H	A	G	N	S	G	G	A	L
Pa D	M	F	G	L	D	L	F	D	G	F	K	A	Q	D	K	E	Q	R	-	Y	L	G	D	A	L	T	D	R	A	I	N	I	I	R	R	H	N	A	S	Q	P	L	F	L	H	F	A	H	N	A	P	H	A	G	N	S	G	G	A	L
Yc D	M	F	G	L	D	L	F	D	G	L	L	P	Q	N	K	E	Q	R	-	Y	I	V	D	A	L	T	D	R	A	I	D	I	I	K	G	H	N	E	S	V	P	L	F	L	H	V	A	H	N	A	P	H	A	G	N	A	G	G	A	L
Bm D	M	L	G	L	D	L	F	D	N	G	I	P	Q	N	H	E	E	R	-	Y	I	V	D	A	L	T	D	R	A	V	K	I	I	H	Q	H	N	T	S	R	P	L	F	L	H	I	T	H	N	A	P	H	A	G	N	A	G	G	A	L
Sf D	M	F	G	L	D	L	Y	D	D	E	I	P	Q	E	T	E	H	R	-	Y	I	V	D	A	L	T	D	N	A	V	K	I	I	Q	Y	H	N	T	S	Q	P	L	F	L	H	V	T	H	N	A	P	H	A	A	N	A	G	A	A	L
Px B	L	F	G	L	D	L	Y	S	D	I	T	P	A	P	E	P	Q	G	-	H	L	T	D	V	L	T	D	H	A	V	Q	L	I	R	S	H	D	S	A	R	P	L	F	L	Q	V	S	H	A	A	P	H	A	G	G	G	M	V	S	L
Yc B	Y	F	G	L	D	L	Y	E	N	L	N	P	V	E	D	T	K	G	-	H	L	S	D	V	L	T	D	R	A	V	Q	L	I	R	E	H	D	T	S	E	P	L	Y	L	Q	L	S	H	A	A	P	H	A	G	G	G	L	V	S	L
Bm B	F	F	G	L	D	L	F	E	N	T	H	P	V	D	N	Q	T	G	-	H	L	A	D	I	I	T	D	Q	A	I	Q	I	I	R	N	H	D	I	N	K	P	L	Y	L	H	V	S	H	A	A	P	H	A	G	G	G	L	V	H	L
Sf B	F	Y	G	L	D	L	F	N	N	L	E	P	V	E	D	Q	R	G	-	H	L	T	E	V	L	T	D	R	A	V	K	L	I	R	S	H	D	P	S	V	P	L	Y	L	H	I	S	H	A	A	P	H	V	G	G	G	F	V	N	L
Bm A	-	-	G	T	D	F	R	R	G	F	E	V	A	H	D	L	F	G	V	Y	A	T	D	V	Y	T	D	E	A	I	K	V	V	N	S	H	N	K	S	E	P	L	F	L	M	L	A	H	S	A	V	H	S	G	N	P	Y	E	P	I
Sf A	-	-	G	F	D	F	R	R	G	Y	E	V	A	Y	D	L	F	G	K	Y	A	T	D	I	Y	T	N	E	A	V	K	L	V	E	A	H	N	K	S	E	P	L	F	L	M	V	A	H	S	A	M	H	S	G	N	P	S	E	F	L
			*			:			.											:			:			.			:			:	:			*	:				*	*	:	*		.	:	*		*		*								:

		199	200	201	202	203	204	205	206	207	208	209	210	211	212	213	214	215	216	217	218	219	220	221	222	223	224	225	226	227	228	229	230	231	232	233	234	235	236	237	238	239	240	241	242	243	244	245	246	247	248	249	250	251	252	253	254	255	256	257
	241	242	243	244	245	246	247	248	249	250	251	252	253	254	255	256	257	258	259	260	261	262	263	264	265	266	267	268	269	270	271	272	273	274	275	276	277	278	279	280	281	282	283	284	285	286	287	288	289	290	291	292	293	294	295	296	297	298	299	300
Px GSS1	Q	A	P	P	E	E	V	R	A	Q	R	H	V	E	L	H	P	R	R	I	F	A	A	M	V	K	K	L	D	D	S	I	G	E	I	V	A	T	L	E	K	K	G	M	L	E	N	T	I	I	**T**	F	**S**	T	D	N	G	A	P	T
Px GSS2	Q	A	P	P	E	E	V	R	A	Q	R	H	V	E	L	H	P	R	R	I	F	A	A	M	V	K	K	L	D	D	S	I	G	E	I	V	A	T	L	E	K	K	G	M	L	E	N	T	I	I	**T**	F	**S**	T	D	N	G	A	P	T
Pa GSS1/2	Q	A	P	P	E	E	V	R	A	Q	R	H	V	E	L	H	P	R	R	I	F	A	A	M	V	K	K	L	D	D	S	I	G	E	I	V	A	T	L	E	K	K	G	M	L	E	N	T	I	I	**T**	F	**S**	T	D	N	G	A	P	T
Px GSS3	Q	A	P	P	E	E	V	R	A	Q	R	H	V	E	L	H	P	R	R	I	F	A	A	M	V	K	K	L	D	D	S	V	G	D	**M**	V	A	T	L	K	E	**Q**	G	M	L	E	N	T	I	I	V	F	V	A	D	N	G	A	P	T
Pa GSS3	Q	A	P	P	E	E	V	R	A	Q	R	H	V	E	L	H	P	R	R	I	F	A	A	M	V	K	K	L	D	D	S	V	G	D	**M**	V	A	T	L	K	E	**Q**	G	M	L	E	N	T	I	I	V	F	V	A	D	N	G	A	P	T
Yc C	Q	A	P	H	K	D	V	R	A	M	R	H	I	E	S	P	E	R	R	I	Y	G	A	M	V	K	K	L	D	D	S	V	G	D	I	V	E	A	L	S	V	K	G	I	L	N	N	T	I	I	V	F	V	A	D	N	G	G	A	A
Bm C1	Q	A	P	P	E	D	V	R	A	M	K	H	V	E	K	T	G	R	R	I	F	A	A	M	M	K	K	L	D	D	G	I	G	E	V	V	E	A	L	H	N	K	G	I	L	D	N	T	V	I	V	F	I	A	D	N	G	G	M	T
Bm C2	Q	A	P	P	E	D	I	R	S	M	K	H	V	E	L	T	G	R	R	T	Y	A	A	M	V	K	K	L	D	E	S	V	G	E	I	V	E	A	L	Y	K	K	Q	M	L	E	N	T	V	I	V	F	I	A	D	N	G	G	M	T
Sf C1	Q	A	P	P	E	V	V	R	E	L	R	Y	I	E	S	P	D	R	R	M	Y	A	A	M	V	K	K	L	D	D	S	V	G	D	I	V	R	A	L	Q	D	K	G	I	L	E	N	T	I	I	V	F	A	A	D	N	G	G	M	T
Sf C2	Q	A	P	P	E	I	I	R	Q	M	R	H	V	E	S	P	E	R	R	I	F	A	A	M	M	K	K	L	D	D	S	V	G	N	I	V	K	A	L	N	E	K	G	I	I	H	N	T	I	V	T	F	I	S	D	N	G	G	M	S
Px D	Q	P	P	L	Y	T	T	V	K	N	R	H	I	A	N	S	D	R	R	L	Y	A	D	I	V	T	H	L	D	R	C	V	G	Q	V	V	R	A	L	A	D	K	N	I	L	D	N	T	I	I	I	F	A	S	D	N	G	A	P	T
Pa D	Q	P	P	L	Y	T	T	V	K	N	M	H	I	A	N	S	D	R	R	L	Y	A	D	I	V	T	H	L	D	R	C	V	G	Q	V	V	R	A	L	A	D	K	N	I	L	D	N	T	I	I	I	F	A	S	D	N	G	A	P	T
Yc D	-	P	R	L	Y	S	T	V	K	N	R	H	V	A	N	S	D	R	R	L	Y	A	D	I	V	T	H	L	D	Q	S	I	G	E	L	V	R	A	L	A	Y	K	N	I	L	H	D	T	L	I	I	F	A	S	D	N	G	A	P	T
Bm D	Q	P	P	L	F	K	T	V	Q	S	K	H	I	A	N	S	N	R	R	L	Y	A	E	I	V	T	H	V	D	Q	S	I	G	R	I	V	V	A	L	A	E	K	G	I	L	D	D	T	I	I	V	F	A	S	D	N	G	S	P	T
Sf D	Q	P	P	L	Y	S	P	V	K	N	D	Y	I	A	N	S	N	R	R	L	Y	S	E	V	V	K	Q	I	D	R	S	V	G	K	I	V	E	A	L	G	D	K	G	I	L	E	D	T	I	I	V	F	V	S	D	N	G	A	P	T
Px B	Q	P	P	P	G	A	V	A	W	N	H	H	V	A	H	S	A	R	R	L	Y	A	G	M	V	T	S	L	D	R	S	I	G	D	I	V	A	A	L	A	D	S	K	M	L	Q	N	T	I	I	A	F	V	S	D	N	G	A	A	P
Yc B	Q	A	A	P	A	S	I	D	S	N	D	H	I	A	H	S	A	R	R	L	Y	A	G	M	V	T	Q	L	D	K	S	V	G	H	I	M	A	V	L	A	E	K	E	I	L	Q	N	T	I	V	V	F	I	S	D	N	G	A	A	P
Bm B	Q	T	P	E	D	T	I	N	A	N	D	H	I	A	H	S	A	R	R	L	Y	A	G	M	V	T	G	L	D	K	S	I	G	N	I	I	A	A	L	A	E	R	E	I	L	D	N	T	L	I	I	F	V	S	D	N	G	A	A	P
Sf B	Q	T	P	E	D	L	I	A	V	N	D	H	I	A	H	S	P	R	R	L	Y	A	G	L	V	T	G	L	D	R	S	V	G	H	V	I	S	A	L	A	E	R	E	L	L	N	N	T	I	V	V	L	I	S	D	N	G	A	A	S
Bm A	R	A	P	Q	K	L	I	D	A	F	K	Y	I	D	D	S	A	R	Q	K	F	A	A	V	L	S	K	L	D	E	S	V	G	K	V	V	K	A	L	H	T	R	G	L	L	E	N	S	I	V	V	F	S	T	D	N	G	G	P	A
Sf A	R	A	P	D	S	V	I	D	K	F	S	H	I	K	D	S	Q	R	R	K	Y	A	A	M	L	T	K	L	D	E	S	V	G	K	V	V	L	S	L	K	Q	K	G	L	L	E	N	T	I	I	L	F	S	S	D	N	G	G	P	A
		.											:					*	.		:	.		:	:	.		:	*			:	*		:				*					:	:		:	:	:	:		:		:	*	*	*	.		.

	258	259	260	261	262	263	264	265	266	267	268	269	270	271	272	273	274	275	276	277	278	279	280	281	282	283	284	285	286	287	288	289	290	291	292	293					294	295	296						297	298	299	300	301	302	303	304	305	306	307	308
	301	302	303	304	305	306	307	308	309	310	311	312	313	314	315	316	317	318	319	320	321	322	323	324	325	326	327	328	329	330	331	332	333	334	335	336	337	338	339	340	341	342	343	344	345	346	347	348	349	350	351	352	353	354	355	356	357	358	359	360
Px GSS1	V	G	L	G	A	N	S	G	S	N	Y	P	L	R	G	V	K	K	S	P	W	E	G	G	I	R	**G**	N	A	**M**	I	W	A	G	P	E	V	A	P	G	N	A	W	R	G	K	V	Y	D	G	N	M	H	A	A	D	W	**V**	P	T
Px GSS2	V	G	L	G	A	N	S	G	S	N	Y	P	L	R	G	V	K	K	S	P	W	E	G	G	I	R	**G**	N	A	**M**	I	W	A	G	P	E	V	A	P	G	N	S	W	R	G	N	V	Y	D	G	N	M	H	A	A	D	W	**V**	P	T
Pa GSS1/2	V	G	L	G	A	N	S	G	S	N	Y	P	L	R	G	V	K	K	S	P	W	E	G	G	I	R	**G**	N	A	**M**	I	W	A	G	P	E	V	A	P	G	N	E	W	R	G	K	V	Y	D	G	N	M	H	A	A	D	W	**V**	P	T
Px GSS3	V	G	N	G	A	N	S	G	S	N	Y	P	L	R	G	V	K	G	S	P	W	E	G	G	I	R	V	D	A	L	**V**	W	A	G	P	E	V	A	E	G	N	D	W	R	G	N	I	F	Q	G	**K**	M	H	A	**S**	D	W	L	P	T
Pa GSS3	V	G	N	G	A	N	S	G	S	N	Y	P	L	R	G	V	K	G	S	P	W	E	G	G	I	R	V	D	G	L	**V**	W	A	G	P	E	V	V	E	G	N	D	W	R	G	N	I	F	Q	G	**K**	M	H	A	**S**	D	W	L	P	T
Yc C	T	G	Q	S	V	N	Y	A	S	N	L	P	L	R	G	S	K	F	S	A	F	E	G	G	T	R	V	I	G	L	I	W	S	A	N	L	-	-	T	S	K	V	W	-	-	-	-	-	S	G	Y	I	H	A	A	D	W	V	P	T
Bm C1	S	G	F	K	M	N	Y	A	S	N	W	P	L	R	G	L	K	M	T	P	F	E	G	G	V	R	V	P	G	L	I	W	S	Q	D	F	K	T	P	Q	N	L	W	-	-	-	-	-	N	G	Y	I	H	V	T	D	W	L	P	I
Bm C2	S	G	V	A	M	N	F	A	S	N	W	P	L	R	G	L	K	M	S	P	F	E	G	G	V	R	A	T	G	L	V	W	S	S	S	F	N	N	S	N	H	L	W	-	-	-	-	-	N	G	F	I	H	V	T	D	W	L	P	I
Sf C1	T	G	L	F	A	N	Y	A	S	N	Y	P	L	R	G	I	K	M	S	P	Y	E	G	G	V	R	V	V	G	L	L	W	S	A	N	L	N	N	T	N	H	Y	W	-	-	-	-	-	D	G	Y	M	H	V	T	D	W	L	P	T
Sf C2	Y	G	Q	Y	N	N	F	A	S	N	H	P	F	R	G	I	K	L	T	P	Y	E	G	G	V	R	V	A	G	L	L	W	S	T	H	L	N	N	T	N	H	Y	W	-	-	-	-	-	D	G	Y	M	H	V	T	D	W	L	P	T
Px D	V	G	P	L	G	N	W	G	I	N	L	P	F	R	G	K	K	Q	T	P	W	E	G	G	V	R	V	P	A	L	I	W	H	S	S	I	-	-	K	P	G	V	W	-	-	-	-	-	D	G	L	M	H	I	T	D	W	L	P	T
Pa D	V	G	P	L	G	N	W	G	I	N	L	P	F	R	G	K	K	L	T	P	W	E	G	G	V	R	V	P	A	L	I	W	H	S	S	I	-	-	K	P	G	V	W	-	-	-	-	-	D	G	L	M	H	I	T	D	W	L	P	T
Yc D	V	G	A	L	R	N	W	G	S	N	L	P	F	R	G	K	K	M	T	P	W	E	G	G	V	R	V	P	A	F	I	W	H	S	S	L	-	-	K	P	T	V	W	-	-	-	-	-	D	G	L	M	H	I	T	D	W	L	P	T
Bm D	V	G	R	L	Q	N	W	G	S	N	L	P	F	R	G	T	K	W	S	P	W	E	G	G	V	R	V	P	A	F	L	W	H	S	S	L	-	-	S	P	R	V	W	-	-	-	-	-	N	G	L	M	H	I	T	D	W	L	P	T
Sf D	V	G	E	L	R	N	W	G	V	N	L	P	L	R	G	K	K	N	T	P	W	E	G	A	V	R	V	P	A	F	I	W	Q	A	S	L	-	-	R	P	K	V	W	-	-	-	-	-	Q	G	L	M	H	I	S	D	W	L	P	T
Px B	V	G	K	T	Q	N	Y	G	T	N	L	P	L	R	G	L	K	G	T	P	F	D	G	G	V	R	S	A	A	V	L	W	H	P	E	L	-	-	E	A	D	S	W	-	-	-	-	-	E	G	L	F	H	V	T	D	W	V	P	T
Yc B	V	G	P	T	Q	N	F	G	S	N	L	P	F	R	G	C	K	G	T	P	W	E	G	G	V	R	A	S	A	L	L	W	H	A	G	L	-	-	V	S	E	I	W	-	-	-	-	-	H	G	L	F	H	V	T	D	W	L	P	T
Bm B	V	G	A	T	Q	N	F	G	S	N	L	P	L	R	G	T	K	G	R	P	W	E	G	A	I	R	S	I	A	I	V	W	H	A	S	I	-	-	T	P	Q	I	W	-	-	-	-	-	N	G	L	F	H	V	T	D	W	L	P	T
Sf B	T	G	L	N	R	N	F	G	S	N	L	P	L	R	G	T	K	G	T	P	W	E	G	A	V	R	T	T	A	V	I	W	H	A	A	L	-	-	A	S	K	I	W	-	-	-	-	-	P	G	L	F	H	V	T	D	W	M	P	T
Bm A	A	G	F	N	D	N	A	A	S	N	Y	P	L	K	G	V	K	N	T	L	W	E	G	G	V	R	G	A	G	F	L	W	S	P	L	L	D	S	K	A	R	V	A	-	-	-	-	-	Y	Q	K	M	H	I	S	D	W	L	P	T
Sf A	A	G	F	N	D	N	A	A	S	N	Y	P	L	K	G	V	K	N	T	L	W	E	G	G	V	R	G	A	A	L	L	W	S	P	L	I	K	Q	P	S	R	V	A	-	-	-	-	-	N	Q	T	F	H	I	A	D	W	L	P	T
		*				*		.		*		*	:	.	*		*				:	:	*	.		*			.	.	:	*																				.	:			*	*	:	*	

	309	310	311	312	313	314	315	316	317				318	319	320	321	322	323	324	325	326	327	328	329	330	331	332	333	334		335		336	337	338	339	340		341	342	343	344			345	346	347	348	349	350	351	352	353	354	355	356	357	358	359	360
	361	362	363	364	365	366	367	368	369	370	371	372	373	374	375	376	377	378	379	380	381	382	383	384	385	386	387	388	389	390	391	392	393	394	395	396	397	398	399	400	401	402	403	404	405	406	407	408	409	410	411	412	413	414	415	416	417	418	419	420
Px GSS1	L	L	E	A	I	G	E	K	I	P	-	-	A	G	L	D	G	I	P	M	W	S	H	I	I	E	N	K	P	-	S	P	R	T	E	I	F	-	E	I	D	D	-	-	Y	F	N	H	S	S	V	T	L	G	R	H	K	L	V	K
Px GSS2	L	L	E	A	V	G	E	K	I	P	-	-	A	G	L	D	G	I	P	M	W	S	H	T	I	E	N	K	P	-	S	P	R	T	E	I	F	-	E	I	D	D	-	-	Y	F	N	H	S	S	V	T	L	G	R	H	K	L	I	K
Pa GSS1/2	L	L	Q	A	I	G	E	V	V	P	-	-	K	N	L	D	G	F	P	M	W	N	E	I	I	E	N	K	P	-	S	P	R	N	E	I	F	-	E	I	D	D	-	-	Y	F	N	H	S	S	V	T	L	G	R	H	K	L	V	K
Px GSS3	L	L	E	A	I	G	E	K	P	P	-	-	T	G	I	D	G	I	P	Q	W	K	H	I	L	E	N	Q	P	-	T	-	R	Q	E	I	F	-	E	I	D	D	-	-	F	T	G	Y	S	S	I	T	L	G	R	H	K	L	I	V
Pa GSS3	L	L	E	A	I	G	E	Q	P	P	-	-	T	G	I	D	G	I	P	Q	W	Q	H	I	L	E	N	Q	P	-	T	-	R	Q	E	I	F	-	E	I	D	D	-	-	F	T	G	Y	S	S	I	T	L	G	R	H	K	L	I	V
Yc C	I	L	G	A	V	N	G	Q	I	P	-	-	E	G	I	D	G	I	N	L	W	K	N	I	T	D	N	T	D	-	S	L	R	E	E	M	F	-	E	I	D	D	-	-	Y	T	G	Y	A	A	I	V	S	G	D	Y	K	L	I	H
Bm C1	F	M	N	I	A	G	A	A	P	P	-	-	N	N	I	S	G	I	D	L	W	N	S	I	K	A	N	E	D	-	S	S	R	Q	E	M	Y	-	E	I	D	D	-	-	R	T	G	F	A	S	V	I	Y	G	D	F	K	L	V	T
Bm C2	F	L	R	I	A	G	S	E	V	P	-	-	L	D	I	N	G	V	D	L	W	D	D	I	N	T	K	D	N	-	S	R	R	D	E	I	Y	-	E	I	D	D	-	-	Y	T	G	F	A	A	I	I	S	Q	D	F	K	L	V	S
Sf C1	L	L	S	A	A	G	V	E	P	P	-	-	T	D	I	D	G	V	D	Q	W	E	S	I	N	S	N	T	A	-	S	Q	R	K	T	M	V	-	E	I	D	D	-	-	Y	T	G	Y	A	S	I	T	Y	G	E	Y	K	M	I	T
Sf C2	L	L	S	A	A	G	V	E	P	P	-	-	P	N	I	D	G	I	D	H	W	K	N	I	N	S	N	L	P	-	S	E	R	E	T	M	Y	-	E	I	D	D	-	-	T	S	G	Y	A	S	A	I	Y	G	D	Y	K	L	V	T
Px D	L	T	A	A	A	G	G	D	A	I	K	-	K	P	I	D	G	V	N	Q	W	N	S	I	I	A	H	S	Q	-	S	P	R	K	E	V	L	I	S	V	E	D	S	E	T	T	M	Y	A	A	Y	R	A	G	D	Y	K	I	V	V
Pa D	L	T	A	A	A	G	G	D	A	I	K	-	N	P	I	D	G	V	N	Q	W	N	S	I	I	A	H	G	Q	-	S	P	R	K	E	V	L	I	S	V	E	D	S	E	T	T	M	Y	A	A	Y	R	A	G	D	Y	K	I	V	V
Yc D	L	A	A	A	A	G	S	S	I	D	-	-	K	E	L	D	G	V	N	Q	W	E	S	I	S	Q	D	G	A	-	S	N	R	N	E	V	L	I	A	L	E	D	S	D	M	N	R	Y	A	A	Y	R	A	G	D	Y	K	V	I	I
Bm D	L	V	A	A	A	G	G	K	V	T	-	-	K	S	I	D	G	V	N	Q	W	D	A	I	L	Q	D	G	E	-	S	K	R	K	E	V	L	I	A	L	E	D	S	N	A	N	M	Y	A	A	Y	R	A	G	D	Y	K	V	I	V
Sf D	L	V	T	A	A	G	G	R	A	P	-	-	T	G	I	D	G	V	N	Q	W	D	S	I	M	F	D	G	V	-	S	K	R	K	E	V	L	I	T	I	E	D	S	N	T	N	A	W	A	S	Y	R	A	G	D	Y	K	I	I	V
Px B	L	V	G	A	A	G	G	N	V	T	-	-	T	E	I	D	G	V	N	Q	W	D	A	I	R	K	G	E	V	-	S	A	R	N	D	M	L	I	S	I	D	D	-	-	L	N	G	W	A	A	I	R	E	G	D	Y	K	L	V	I
Yc B	L	V	A	A	A	G	G	N	V	-	-	-	S	K	V	D	G	V	N	Q	W	S	A	L	K	D	N	T	Q	-	S	T	R	E	S	V	L	I	S	I	D	D	-	-	L	N	G	W	A	A	Y	R	E	G	D	F	K	F	V	V
Bm B	L	V	A	A	A	G	G	K	A	P	-	-	T	G	I	D	G	V	N	Q	W	N	A	L	T	K	D	E	D	-	S	S	R	K	R	L	L	L	T	V	D	D	-	-	L	N	G	W	A	A	Y	R	D	G	D	F	K	I	I	I
Sf B	L	I	A	A	A	G	G	E	Y	N	-	-	G	T	I	D	G	I	N	Q	W	N	T	L	T	K	N	D	T	-	S	P	R	Q	D	I	L	I	T	T	D	D	-	-	L	N	E	Y	A	A	F	R	E	G	N	Y	K	I	I	I
Bm A	L	Y	S	A	A	G	G	D	L	S	V	L	E	N	L	D	G	V	N	Q	W	D	A	L	S	K	N	T	E	-	S	P	R	T	S	V	L	H	N	I	D	D	-	-	I	W	G	I	A	A	L	T	V	D	K	Y	K	L	I	K
Sf A	L	Y	S	A	A	G	G	E	T	S	V	F	K	N	L	D	G	F	D	L	W	E	A	L	S	N	D	Q	P	-	S	Q	R	T	K	I	V	H	N	I	D	D	-	-	I	F	G	S	A	S	I	T	V	D	N	W	K	L	H	K
	:					.									:	.	*	.			*										.		*			:					:	*							:	:							*	.		

	361	362	363		364	365	366	367	368	369	370	371	372	373	374										375	376	377	378	379	380	381	382	383	384	385	386	387	388	389		390	391	392	393	394	395	396	397	398	399	400	401	402	403						
	421	422	423	424	425	426	427	428	429	430	431	432	433	434	435	436	437	438	439	440	441	442	443	444	445	446	447	448	449	450	451	452	453	454	455	456	457	458	459	460	461	462	463	464	465	466	467	468	469	470	471	472	473	474	475	476	477	478	479	480
Px GSS1	G	T	I	-	D	E	S	L	S	K	H	Y	G	E	D	L	R	G	I	I	G	T	P	P	D	Y	K	Q	K	L	R	D	S	K	A	W	E	S	L	-	E	T	I	G	I	P	L	D	A	D	V	M	A	D	-	R	D	E	A	I
Px GSS2	G	T	I	-	E	E	S	L	S	K	H	Y	G	E	D	L	R	G	I	I	G	T	P	P	D	Y	K	Q	K	L	R	D	S	K	A	W	E	S	L	-	E	T	I	G	I	P	L	D	A	D	V	M	A	D	-	R	D	E	A	I
Pa GSS1/2	G	T	I	-	D	E	S	L	S	K	H	Y	G	A	D	L	R	G	I	I	G	T	P	P	D	Y	K	Q	K	L	R	D	S	K	A	W	E	S	L	-	E	T	I	G	I	P	L	D	A	D	V	M	A	D	-	R	D	E	A	I
Px GSS3	G	T	V	-	N	A	A	Y	N	L	H	R	G	G	D	L	R	G	I	I	G	Q	P	P	N	Y	Q	Q	A	V	L	N	S	K	A	Y	A	A	I	-	T	A	A	G	V	P	I	D	V	E	H	L	E	E	N	K	E	K	A	T
Pa GSS3	G	T	V	-	N	P	P	Y	N	L	H	R	G	G	D	L	R	G	I	I	G	Q	P	P	N	Y	Q	Q	A	V	R	N	S	K	A	Y	A	A	I	-	T	A	A	G	V	P	I	D	V	E	H	L	E	E	N	K	E	K	A	T
Yc C	G	A	V	-	P	S	T	Y	S	N	Y	Y	N	E	D	L	T	G	V	I	G	T	E	P	S	Y	S	E	A	L	T	S	S	K	V	Y	G	V	L	-	K	N	L	G	K	C	L	K	V	D	T	W	T	T	-	Q	N	D	T	V
Bm C1	G	H	V	-	T	E	E	Y	S	D	Y	E	G	G	D	L	R	G	I	I	G	E	P	P	S	Y	N	D	A	I	E	K	S	K	V	Y	G	V	L	-	R	K	I	G	K	P	V	K	K	I	D	Y	N	L	-	R	K	Q	I	E
Bm C2	G	Q	V	-	T	L	E	Y	S	N	Y	Q	G	E	N	L	R	G	M	I	G	D	P	P	S	Y	A	D	S	V	Q	K	S	K	T	Y	R	I	L	-	K	N	I	G	K	P	F	K	K	E	D	V	N	L	-	R	R	N	I	R
Sf C1	G	E	P	-	I	Q	S	H	S	E	Y	L	G	G	D	L	R	G	L	I	G	S	P	P	S	Y	I	D	T	I	A	D	S	K	V	F	G	A	L	-	H	S	I	G	R	T	F	D	M	D	F	L	G	L	-	R	N	K	T	I
Sf C2	G	D	V	-	D	K	G	Y	G	I	H	Q	G	N	N	L	T	G	I	I	G	K	P	P	S	Y	V	D	A	I	L	D	S	T	V	Y	S	V	L	-	D	S	I	G	L	T	F	D	I	A	N	L	N	R	-	R	K	I	T	T
Px D	G	N	V	-	T	G	V	S	N	G	Y	Y	G	A	E	F	M	I	N	K	K	T	P	P	D	Y	Y	S	V	L	K	D	C	E	V	A	N	V	L	-	Q	T	L	G	L	H	L	D	K	Q	E	V	E	E	L	R	L	A	A	R
Pa D	G	N	V	-	T	G	V	S	N	G	Y	Y	G	A	E	F	M	I	N	K	K	T	P	P	D	Y	Y	S	V	L	K	D	S	E	A	A	N	V	L	-	Q	T	L	G	L	H	L	D	K	Q	E	V	E	E	L	R	L	A	A	R
Yc D	G	N	V	-	S	G	L	S	N	G	F	Y	G	S	E	L	M	V	N	K	E	S	P	P	D	Y	H	T	I	L	R	N	C	E	A	A	K	A	L	-	N	S	I	G	L	F	T	T	R	E	Q	I	D	T	S	R	L	A	A	T
Bm D	G	N	V	-	T	G	L	N	N	N	V	Y	G	T	E	L	L	I	N	K	F	A	P	P	P	Y	F	S	T	L	K	S	C	E	V	A	Q	M	F	-	E	Y	T	G	R	Y	L	D	F	D	V	V	Q	N	M	R	Q	A	A	T
Sf D	G	N	V	-	T	G	V	S	N	G	Y	Y	G	A	E	L	M	A	N	K	M	P	P	P	E	Y	F	P	A	L	G	N	S	V	V	T	R	V	F	-	G	D	I	G	R	Y	L	D	F	D	V	V	R	A	M	R	K	A	A	T
Px B	G	I	V	-	D	P	S	L	S	D	Y	Y	G	E	S	L	M	G	V	R	F	K	S	P	S	Y	E	G	T	L	L	D	C	E	T	A	K	V	I	K	E	S	L	A	K	D	I	D	I	N	V	A	I	I	R	R	N	E	S	T
Yc B	G	H	L	-	N	N	E	A	S	K	Y	H	G	S	K	L	M	E	L	R	L	Q	G	P	A	Y	E	N	S	L	L	E	C	E	T	A	R	I	F	K	E	F	L	E	R	D	L	S	I	D	N	V	I	M	K	R	N	D	S	S
Bm B	G	D	V	-	D	K	E	T	S	V	Y	L	G	N	E	F	K	E	L	R	L	P	V	H	L	Y	E	S	T	L	L	S	S	E	A	A	H	V	F	K	E	I	L	D	L	Y	L	D	L	D	E	I	F	H	K	R	D	K	L	S
Sf B	G	K	V	-	E	P	Q	Q	S	D	Y	Y	G	M	E	L	K	K	S	R	M	P	G	F	L	Y	E	N	V	L	L	S	S	D	T	A	R	V	F	R	E	T	L	F	I	N	I	D	L	E	M	A	A	T	K	R	N	Y	S	D
Bm A	G	T	I	Y	K	G	V	W	D	N	W	Y	G	P	S	-	-	-	-	-	G	R	E	G	A	Y	N	A	S	L	L	Y	D	S	H	A	G	R	I	L	D	K	L	N	L	M	P	P	K	E	K	V	M	E	L	R	D	E	A	T
Sf A	G	T	N	Y	N	G	T	W	D	Y	W	Y	G	P	A	-	-	-	-	-	G	R	D	G	L	Y	N	F	D	L	L	Y	K	S	Y	A	G	V	A	I	N	N	I	T	A	L	P	D	K	E	R	I	I	S	I	R	D	A	S	T
	*								.				.													*																														.				

																		404	405	406		407	408	409		410	411	412	413	414	415	416	417	418	419	420	421	422	423	424	425	426	427	428	429	430	431	432	433	434	435	436	437	438	439					
	481	482	483	484	485	486	487	488	489	490	491	492	493	494	495	496	497	498	499	500	501	502	503	504	505	506	507	508	509	510	511	512	513	514	515	516	517	518	519	520	521	522	523	524	525	526	527	528	529	530	531	532	533	534	535	536	537	538	539	540
Px GSS1	V	-	-	-	-	-	T	C	-	G	-	N	V	V	P	K	P	C	S	P	S	A	E	S	W	C	L	Y	D	I	I	E	D	P	C	E	L	R	D	L	S	E	E	L	P	Q	L	A	Q	I	L	L	Y	R	L	E	Q	E	E	A
Px GSS2	V	-	-	-	-	-	T	C	-	G	-	D	V	V	P	K	P	C	S	P	T	A	E	S	W	C	L	Y	D	I	I	A	D	P	C	E	L	R	D	L	S	E	E	L	P	Q	L	A	Q	I	L	L	Y	R	L	E	Q	E	E	A
Pa GSS1/2	I	-	-	-	-	-	T	C	-	G	-	D	V	E	P	K	P	C	S	P	S	A	D	S	W	C	L	Y	D	I	I	E	D	P	C	E	L	R	D	L	S	E	E	L	P	Q	L	A	Q	I	L	L	Y	R	L	E	Q	E	E	A
Px GSS3	V	-	-	-	-	-	T	C	-	G	-	E	G	V	A	K	E	C	I	P	T	A	D	K	W	C	L	Y	D	I	I	D	D	P	C	E	Y	R	D	L	S	D	S	L	P	G	L	A	E	V	L	R	F	R	L	A	Q	E	E	E
Pa GSS3	V	-	-	-	-	-	T	C	-	G	-	K	G	V	A	K	E	C	I	P	S	A	E	K	W	C	L	Y	D	I	I	D	D	P	C	E	Y	R	D	L	S	E	S	L	P	G	L	A	Q	V	L	R	F	R	L	S	Q	E	E	E
Yc C	I	-	-	-	-	-	D	C	-	G	-	S	K	V	F	A	E	C	Q	P	T	V	A	K	Y	C	L	Y	N	I	K	E	D	P	C	E	M	E	D	L	S	S	T	E	T	K	L	A	E	T	L	Y	A	R	L	Q	K	E	Y	E
Bm C1	V	-	-	-	-	-	K	C	-	D	R	K	D	T	D	E	I	C	Y	P	N	K	D	K	I	C	L	Y	N	I	K	E	D	P	C	E	T	R	D	V	S	K	E	N	P	D	I	V	V	N	M	L	Q	L	L	K	V	E	L	T
Bm C2	V	-	-	-	-	-	K	C	-	N	-	Q	E	I	D	K	I	C	Y	P	D	K	N	K	F	C	L	Y	N	I	K	E	D	P	C	E	T	N	D	L	S	A	T	Y	P	D	I	A	E	S	M	L	K	R	L	H	I	E	I	S
Sf C1	I	-	-	-	-	-	N	C	-	D	-	A	S	A	T	S	I	C	Y	P	S	N	D	T	V	C	L	F	N	I	I	E	D	P	C	E	T	T	D	L	S	S	T	Y	P	E	L	V	T	T	M	Q	A	L	L	D	I	E	M	S
Sf C2	V	-	-	-	-	-	K	C	-	V	L	P	S	P	V	T	L	C	H	P	S	K	E	T	S	C	L	F	N	I	K	E	D	P	C	E	T	T	N	L	S	A	T	Y	P	D	L	V	S	R	M	Q	A	E	L	E	K	E	V	A
Px D	V	-	-	-	-	-	K	Q	L	D	G	V	R	N	A	T	P	C	E	P	T	P	T	R	G	C	L	Y	N	V	R	R	D	P	M	E	A	H	D	L	W	D	Q	A	R	R	I	A	S	L	L	T	S	R	L	Q	E	L	W	A
Pa D	V	-	-	-	-	-	K	Q	L	D	G	V	R	N	A	T	P	C	E	P	T	P	T	R	G	C	L	Y	N	V	R	R	D	P	M	E	A	H	D	L	W	D	Q	A	R	K	I	A	S	L	L	T	C	R	L	Q	E	L	W	A
Yc D	I	-	-	-	-	-	R	Q	Q	D	V	Q	S	D	V	R	N	C	E	P	S	P	T	R	G	C	L	Y	N	I	R	R	D	P	M	E	T	H	N	L	W	R	Q	A	S	R	I	S	T	M	M	T	N	R	L	R	E	L	W	G
Bm D	V	-	-	-	-	-	K	Q	I	D	E	I	K	D	A	T	L	C	I	P	S	L	T	R	G	C	L	F	N	L	R	K	D	P	S	E	S	H	D	L	W	Q	E	A	N	K	I	A	M	L	L	T	S	R	L	R	G	L	W	A
Sf D	I	-	-	-	-	-	R	A	D	E	T	K	R	D	A	T	P	C	V	P	T	P	T	R	G	C	L	Y	N	V	R	Q	D	P	T	E	S	R	D	L	W	T	R	A	N	K	I	A	T	L	L	T	S	R	L	R	A	L	W	S
Px B	L	-	-	-	-	-	S	P	A	G	Q	I	N	I	T	D	L	C	I	P	N	S	E	K	G	C	L	F	N	I	I	E	D	P	H	E	A	H	D	L	W	A	Q	L	P	E	L	V	K	R	L	T	L	R	L	R	A	L	W	S
Yc B	I	-	-	-	-	-	I	K	I	V	D	E	E	V	S	E	L	C	I	P	S	Q	A	K	G	C	L	Y	N	I	K	D	D	P	F	E	A	N	D	L	W	N	A	S	P	D	L	V	K	L	L	T	L	K	I	R	S	L	W	A
Bm B	L	-	R	S	-	-	K	H	-	N	I	S	Q	E	F	N	F	C	V	P	S	R	E	S	G	C	L	F	N	I	T	A	D	P	L	E	T	T	N	L	W	P	S	L	P	D	V	V	R	R	M	V	L	R	L	R	Y	Y	W	A
Sf B	L	Y	R	H	I	N	N	T	-	D	S	V	T	N	E	G	L	C	I	P	T	N	A	K	G	C	L	F	D	L	S	V	D	P	A	E	S	K	N	L	W	H	T	H	P	Q	F	V	Q	H	M	V	L	R	L	R	A	Y	W	A
Bm A	V	-	-	-	-	-	K	C	-	N	D	S	I	E	V	I	Q	C	K	P	R	D	A	P	-	C	V	F	N	I	D	E	D	P	C	E	R	R	N	L	A	Q	L	L	P	E	V	L	E	S	L	L	S	E	M	H	K	L	N	V
Sf A	I	-	-	-	-	-	K	C	-	D	N	G	K	P	I	N	E	C	K	P	L	K	A	P	-	C	L	F	D	I	H	G	D	P	C	E	L	K	N	L	A	N	E	K	P	D	V	L	Q	A	L	L	S	E	L	E	R	V	N	A
	:																	*		*						*	:	:	:	:			*	*		*			:	:							.				:				:					

	541	542	543	544	545	546	547	548	549	550	551	552	553	554	555	556	557	558	559	560	561	562	563	564	565	566	567	568	569	570	571	572	573	574	575	576	577	578	579	580	581	582	583	584	585	586	587	588	589	590	591	592	593	594	595	596	597	598	599	600
Px GSS1	K	I	I	P	R	E	-	G	Q	Y	-	-	V	A	D	P	K	S	A	P	K	Y	F	N	Y	T	W	D	A	Y	L	S	V	E	P	Y	S	D	S	E	-	-	-	-	-	-	-	-	-	-	-	-	-	-	-	-	-	-	-	-
Px GSS2	K	I	I	P	R	D	-	G	Q	F	-	-	V	A	D	P	R	A	A	P	K	Y	F	N	Y	T	W	D	S	Y	L	S	V	E	P	Y	S	D	S	E	-	-	-	-	-	-	-	-	-	-	-	-	-	-	-	-	-	-	-	-
Pa GSS1/2	K	I	I	P	R	E	-	S	Q	Y	-	-	I	A	D	P	R	S	A	P	K	Y	F	N	Y	T	W	D	A	Y	L	S	V	E	P	Y	T	D	S	A	-	-	-	-	-	-	-	-	-	-	-	-	-	-	-	-	-	-	-	-
Px GSS3	R	V	V	P	R	T	-	D	K	N	D	P	I	G	S	E	R	A	M	S	K	N	F	N	Y	T	W	E	T	F	M	D	L	E	P	Y	T	A	-	-	-	-	-	-	-	-	-	-	-	-	-	-	-	-	-	-	-	-	-	-
Pa GSS3	R	V	V	P	R	T	-	D	Q	N	D	P	I	G	S	E	R	A	M	P	K	N	F	N	Y	T	W	E	T	F	M	D	L	E	P	Y	T	A	-	-	-	-	-	-	-	-	-	-	-	-	-	-	-	-	-	-	-	-	-	-
Yc C	K	R	I	P	R	T	-	V	P	L	-	-	Y	R	D	P	L	S	R	P	S	Y	Y	N	Y	T	W	T	A	F	I	D	V	G	T	S	I	K	S	K	N	Q	I	N	K	K	-	-	-	-	-	-	-	-	-	-	-	-	-	-
Bm C1	K	T	V	P	R	T	D	P	P	L	-	-	A	S	D	P	R	S	L	P	S	L	H	N	N	T	W	D	T	F	I	D	-	-	-	-	-	-	-	-	-	-	-	-	-	-	-	-	-	-	-	-	-	-	-	-	-	-	-	-
Bm C2	K	T	I	P	R	T	-	V	P	L	-	-	Y	R	N	P	E	S	L	P	R	L	H	N	F	T	W	T	T	F	A	D	N	F	K	-	-	-	-	-	-	-	-	-	-	-	-	-	-	-	-	-	-	-	-	-	-	-	-	-
Sf C1	K	R	I	P	R	I	-	A	P	A	-	-	S	I	D	R	M	S	A	P	S	L	H	N	Y	T	W	D	T	W	I	N	-	-	-	-	-	-	-	-	-	-	-	-	-	-	-	-	-	-	-	-	-	-	-	-	-	-	-	-
Sf C2	K	T	I	F	R	K	-	G	P	V	-	-	F	R	D	P	L	A	A	P	R	R	F	N	Y	T	W	D	V	W	V	K	-	-	-	-	-	-	-	-	-	-	-	-	-	-	-	-	-	-	-	-	-	-	-	-	-	-	-	-
Px D	Q	A	N	K	R	-	-	G	P	T	-	-	A	L	K	A	E	A	D	P	A	N	F	D	Y	T	W	L	P	W	L	S	E	P	K	D	Y	D	Y	N	D	I	N	S	I	L	-	N	Q	R	R	G	R	A	G	L	P	A	N	L
Pa D	Q	A	N	K	R	-	-	G	P	T	-	-	A	L	Q	A	E	A	D	P	A	N	F	D	Y	T	W	L	P	W	L	S	E	P	K	D	Y	D	Y	N	D	I	N	S	I	V	-	N	Q	R	R	S	R	A	G	L	P	A	N	L
Yc D	M	Q	Q	R	R	-	-	G	P	F	-	-	N	L	Q	A	E	A	D	P	A	L	F	D	Y	T	W	M	P	W	L	S	E	T	N	L	L	A	S	I	D	D	N	T	L	T	Y	D	S	L	I	G	K	S	D	A	L	L	E	L
Bm D	M	Q	L	R	R	-	-	G	P	T	-	-	N	L	R	N	E	S	D	P	A	K	F	G	Y	T	W	T	P	Y	V	K	N	K	D	I	T	K	V	P	V	Q	-	-	-	-	-	-	-	K	I	S	N	L	N	L	S	I	D	I
Sf D	S	Q	L	R	R	-	-	G	P	L	-	-	R	L	Q	E	A	A	D	P	A	N	N	H	Y	V	W	H	P	W	I	C	C	N	E	T	R	T	N	S	V	T	N	D	K	K	-	-	-	T	I	D	N	F	D	F	S	F	N	F
Px B	D	M	R	G	R	-	-	H	P	A	-	-	R	P	D	A	R	A	D	P	A	L	H	Q	H	A	W	A	P	W	L	E	D	S	E	P	A	P	L	H	A	P	Q	P	P	E	F	P	L	R	V	T	K	A	E	L	Q	Y	L	V
Yc B	N	I	I	P	R	S	-	V	P	-	-	-	K	L	D	N	R	A	N	P	S	K	K	N	F	I	W	S	P	W	V	K	E	D	E	P	Y	P	Q	T	L	L	T	P	P	L	F	P	L	Q	V	S	K	A	E	L	Q	Y	L	V
Bm B	Q	I	N	P	R	-	-	C	L	V	-	-	K	I	D	K	K	S	D	P	S	L	R	D	F	V	W	S	P	W	L	K	D	N	E	Q	V	A	K	P	I	R	D	R	P	R	F	P	L	K	V	L	V	G	E	L	Q	Y	L	I
Sf B	A	I	N	P	R	H	-	E	P	-	-	-	T	P	D	P	R	A	N	P	A	L	H	D	F	I	W	L	P	W	L	N	N	L	E	N	V	T	K	P	L	L	A	F	P	N	F	P	L	Q	V	S	V	G	E	L	Q	Y	L	V
Bm A	S	A	V	A	P	-	-	N	A	Q	-	-	P	I	D	A	R	G	D	P	Q	Y	W	G	R	V	Y	T	N	F	G	N	Y	E	T	Q	H	G	S	S	V	C	L	-	-	-	-	-	-	-	-	-	-	-	-	-	-	-	-	-
Sf A	T	A	V	P	P	-	-	N	N	A	-	-	D	L	D	T	R	G	D	P	K	Y	W	G	R	V	F	T	N	F	G	D	N	V	S	L	-	-	-	-	-	-	-	-	-	-	-	-	-	-	-	-	-	-	-	-	-	-	-	-
																		.		.							:			:																														

	601	602	603	604	605	606	607	608	609	610	611	612	613	614	615	616	617	618	619	620	621	622	623	624	625	626	627	628	629	630	631	632	633	634	635	636
Px GSS1	-	-	-	-	-	-	-	-	-	-	-	-	-	-	-	-	-	-	-	-	-	-	-	-	-	-	-	-	-	-	-	-	-	-	-	-
Px GSS2	-	-	-	-	-	-	-	-	-	-	-	-	-	-	-	-	-	-	-	-	-	-	-	-	-	-	-	-	-	-	-	-	-	-	-	-
Pa GSS1/2	-	-	-	-	-	-	-	-	-	-	-	-	-	-	-	-	-	-	-	-	-	-	-	-	-	-	-	-	-	-	-	-	-	-	-	-
Px GSS3	-	-	-	-	-	-	-	-	-	-	-	-	-	-	-	-	-	-	-	-	-	-	-	-	-	-	-	-	-	-	-	-	-	-	-	-
Pa GSS3	-	-	-	-	-	-	-	-	-	-	-	-	-	-	-	-	-	-	-	-	-	-	-	-	-	-	-	-	-	-	-	-	-	-	-	-
Yc C	-	-	-	-	-	-	-	-	-	-	-	-	-	-	-	-	-	-	-	-	-	-	-	-	-	-	-	-	-	-	-	-	-	-	-	-
Bm C1	-	-	-	-	-	-	-	-	-	-	-	-	-	-	-	-	-	-	-	-	-	-	-	-	-	-	-	-	-	-	-	-	-	-	-	-
Bm C2	-	-	-	-	-	-	-	-	-	-	-	-	-	-	-	-	-	-	-	-	-	-	-	-	-	-	-	-	-	-	-	-	-	-	-	-
Sf C1	-	-	-	-	-	-	-	-	-	-	-	-	-	-	-	-	-	-	-	-	-	-	-	-	-	-	-	-	-	-	-	-	-	-	-	-
Sf C2	-	-	-	-	-	-	-	-	-	-	-	-	-	-	-	-	-	-	-	-	-	-	-	-	-	-	-	-	-	-	-	-	-	-	-	-
Px D	N	E	R	S	G	D	V	K	Y	S	G	L	R	S	S	S	V	C	E	G	T	S	G	I	R	Q	F	I	C	V	L	Q	N	V	F	-
Pa D	N	E	R	S	G	D	V	K	Y	N	S	L	R	S	S	S	V	C	E	G	T	S	G	I	R	Q	F	I	C	V	L	Q	N	V	F	-
Yc D	G	D	R	R	G	R	I	S	G	N	V	V	G	H	-	-	-	-	-	-	-	-	-	-	-	-	-	-	-	-	-	-	-	-	-	-
Bm D	S	G	N	R	T	S	T	K	D	T	T	K	A	S	A	V	N	C	D	G	I	T	G	I	R	N	F	L	C	L	L	Q	S	V	F	-
Sf D	N	Q	E	R	I	V	N	R	N	T	T	L	A	S	V	I	N	C	D	R	T	T	G	L	R	N	F	L	C	I	L	R	S	I	F	-
Px B	D	I	N	L	D	A	F	K	T	K	I	Y	E	S	I	N	S	M	G	-	-	N	S	F	V	K	S	V	G	S	L	F	S	F	K	-
Yc B	D	L	N	L	G	S	F	K	E	K	M	F	D	Y	I	Q	N	M	G	-	-	D	S	F	V	K	S	V	G	G	L	F	N	F	-	-
Bm B	D	S	K	F	N	N	F	S	E	T	M	S	A	Y	V	K	S	M	A	-	-	D	S	F	L	E	N	V	S	E	L	F	S	F	-	-
Sf B	D	F	N	L	N	A	F	K	E	S	L	T	S	Y	V	K	T	S	A	-	-	D	A	F	I	Q	N	I	A	K	L	F	S	F	-	-
Bm A	-	-	-	-	-	-	-	-	-	-	-	-	-	-	-	-	-	-	-	-	-	-	-	-	-	-	-	-	-	-	-	-	-	-	-	-
Sf A	-	-	-	-	-	-	-	-	-	-	-	-	-	-	-	-	-	-	-	-	-	-	-	-	-	-	-	-	-	-	-	-	-	-	-	-

((PxGSS1, PxGSS2), PaGSS1/2) #1
0.99 < P
0.95 < P ≤ 0.99
0.80 < P ≤ 0.95

(PxGSS3, PaGSS3) #1
0.99 < P
0.95 < P ≤ 0.99
0.80 < P ≤ 0.95

((PxGSS1, PxGSS2), PaGSS1/2 #1)
0.99 < P
0.95 < P ≤ 0.99
0.80 < P ≤ 0.95

(((PxGSS1, PxGSS2), PaGSS), (PxGSS3, PaGSS3)) #1
0.80 < P

not used for PAML

arylsulfatase signatures
conserved arginine

signal peptide

Table S4. Branch-site unrestricted statistical tests for episodic diversification (BUSTED).

Branch	Model	*log* L	# params	AIC$_c$	*p*	Branch set	ω_1	ω_2	ω_3
((((((PxGSS1a, PxGSS1b) #1, (PxGSS2a, PxGSS2b) #2) #3, PaGSS1/2 #4) #5, ((PxGSS3a, PxGSS3b) #6, PaGSS3 #7) #8) #9, YcC #10) #11, ((BmC1 #12, BmC2 #13) #14, (SfC1 #15, SfC2 #16) #17) #18), ((((PxD, PaD), YcD), (BmD, SfD)), ((PxB, YcB), (BmB, SfB))), (BmA, SfA));									
#1	Uconstrained	-21014	69	42168	1.000	Test	0.00 (70.12%)	0.24 (19.92%)	9.52 (9.96%)
						Background	0.01 (74.87%)	0.33 (19.74%)	1.84 (5.39%)
	Constrained	-21014	68	42166		Test	0.00 (70.12%)	0.24 (19.92%)	1.00 (9.96%)
						Background	0.01 (74.95%)	0.34 (19.68%)	1.84 (5.37%)
#2	Uconstrained	-21010	69	42159	**0.010**	Test	0.03 (74.87%)	0.07 (24.18%)	55.11 (0.95%)
						Background	0.01 (75.05%)	0.32 (18.80%)	1.60 (6.15%)
	Constrained	-21015	68	42166		Test	0.00 (24.77%)	0.00 (59.01%)	1.00 (16.22%)
						Background	0.01 (75.09%)	0.32 (18.75%)	1.60 (6.17%)
#3	Uconstrained	-21012	69	42163	0.117	Test	0.08 (13.58%)	0.09 (85.67%)	89.44 (0.74%)
						Background	0.01 (75.78%)	0.33 (17.96%)	1.56 (6.26%)
	Constrained	-21014	68	42166		Test	0.00 (28.48%)	0.00 (51.00%)	1.00 (20.53%)
						Background	0.01 (75.81%)	0.35 (19.03%)	1.84 (5.16%)
#4	Uconstrained	-21003	69	42146	**0.000**	Test	0.00 (15.82%)	0.00 (82.05%)	43.24 (2.13%)
						Background	0.01 (73.08%)	0.23 (16.99%)	1.12 (9.93%)
	Constrained	-21014	68	42165		Test	0.00 (4.71%)	0.00 (80.39%)	1.00 (14.91%)
						Background	0.01 (72.65%)	0.22 (17.19%)	1.11 (10.15%)
#5	Uconstrained	-20970	69	42079	**0.000**	Test	1.00 (56.41%)	1.00 (29.90%)	196.55 (13.69%)
						Background	0.01 (80.02%)	0.55 (19.66%)	122.91 (0.31%)
	Constrained	-21003	68	42143		Test	1.00 (13.69%)	1.00 (56.41%)	1.00 (29.90%)
						Background	0.01 (80.56%)	0.58 (19.12%)	132.77 (0.32%)
#6	Uconstrained	-21010	69	42160	0.623	Test	0.00 (85.89%)	0.01 (12.55%)	4.05 (1.56%)
						Background	0.01 (74.45%)	0.33 (20.07%)	1.92 (5.48%)
	Constrained	-21011	68	42159		Test	0.00 (13.80%)	0.00 (81.59%)	1.00 (4.60%)
						Background	0.01 (74.54%)	0.33 (20.01%)	1.92 (5.45%)
#7	Uconstrained	-21012	69	42164	-	Test	0.05 (100.00%)	0.71 (0.00%)	1.97 (0.00%)
						Background	0.01 (76.04%)	0.39 (19.74%)	2.33 (4.22%)
#8	Uconstrained	-20980	69	42100	**0.000**	Test	1.00 (43.20%)	1.00 (46.74%)	326.40 (10.07%)
						Background	0.01 (80.29%)	0.60 (19.41%)	123.19 (0.30%)
	Constrained	-20996	68	42128		Test	0.00 (64.90%)	1.00 (0.00%)	1.00 (35.10%)
						Background	0.01 (80.56%)	0.61 (19.15%)	133.64 (0.29%)
#9	Uconstrained	-21007	69	42153	1.000	Test	0.01 (88.32%)	1.00 (0.00%)	∞ (11.68%)
						Background	0.00 (65.86%)	0.24 (27.80%)	1.75 (6.34%)
	Constrained	-21007	68	42151		Test	0.00 (88.31%)	0.98 (0.00%)	1.00 (11.68%)
						Background	0.00 (65.76%)	0.23 (27.91%)	1.75 (6.33%)
#10	Uconstrained	-21014	69	42166	0.99	Test	0.00 (71.48%)	0.36 (27.31%)	370.60 (1.22%)
						Background	0.01 (74.71%)	0.30 (18.67%)	1.59 (6.62%)
	Constrained	-21014	68	42164		Test	0.00 (71.85%)	0.37 (28.15%)	1.00 (0.00%)
						Background	0.01 (74.71%)	0.30 (18.76%)	1.61 (6.54%)

Table S5. Adaptive branch-site REL test (aBSREL) for episodic diversification.

Branch	Nucleotide GTR			Baseline MG94xREV			Full adaptive model						
(((((((PxGSS1a, PxGSS1b) #1, (PxGSS2a, PxGSS2b) #2) #3, PaGSS1/2 #4) #5, ((PxGSS3a, PxGSS3b) #6, PaGSS3 #7) #8) #9, YcC #10) #11, ((BmC1 #12, BmC2 #13) #14, (SfC1 #15, SfC2 #16) #17) #18), ((((PxD, PaD), YcD), (BmD, SfD)), ((PxB, YcB), (BmB, SfB))), (BmA, SfA));	**AIC_C**	**log L**	**Parameters**	**AIC_C**	**log L**	**Parameters**	**AIC_C**	**log L**	**Parameters**	**LRT**	**p-value**	**ω distribution over sites**	
#1	46679.8	-23286.8	53	42607.7	-21198.8	104	41948.8	-20799.5	172	0.3	0.3795	$\omega_1 = \infty$ (100%)	
#2	46679.8	-23286.8	53	42607.7	-21198.8	104	41948.8	-20799.5	172	**11.8**	**0.0009**	$\omega_1 = 0.00$ (99%)	$\omega_2 = 55.0$ (1.1%)
#3	46679.8	-23286.8	53	42607.7	-21198.8	104	41948.8	-20799.5	172	0.3	0.3668	$\omega_1 = 0.0595$ (99%)	$\omega_2 = 7.55$ (0.98%)
#4	46679.8	-23286.8	53	42607.7	-21198.8	104	41948.9	-20799.6	172	**16.1**	**0.0001**	$\omega_1 = 0.00$ (98%)	$\omega_2 = 62.9$ (1.8%)
#5	46679.8	-23286.8	53	42607.7	-21198.8	104	41948.8	-20799.6	172	**10.0**	**0.0024**	$\omega_1 = 0.601$ (88%)	$\omega_2 = 93.7$ (12%)
#6	46679.8	-23286.8	53	42607.7	-21198.8	104	41948.9	-20799.6	172	2.0	0.1447	$\omega_1 = 0.000208$ (99%)	$\omega_2 = 7.40$ (1.2%)
#7	46679.8	-23286.8	53	42607.7	-21198.8	104	41948.9	-20799.6	172	0.0	1	$\omega_1 = 0.0959$ (100%)	
#8	46679.8	-23286.8	53	42607.7	-21198.8	104	41949.7	-20800.0	172	**5.0**	**0.0293**	$\omega_1 = 0.0861$ (92%)	$\omega_2 = 25.8$ (7.9%)
#9	46679.8	-23286.8	53	42607.7	-21198.8	104	41948.9	-20799.6	172	0.0	0.5	$\omega_1 = 0.00383$ (90%)	$\omega_2 = 53.1$ (10%)
#10	46679.8	-23286.8	53	42607.7	-21198.8	104	41948.9	-20799.6	172	0.0	0.4779	$\omega_1 = 0.00$ (72%)	$\omega_2 = 1.14$ (28%)

Table S6. Detailed results of McDonald-Kreitman comparisons of *P. xylostella GSS* genes with reconstructed ancestral sequences.

		PxGSS1 (N = 60)					*PxGSS2* (N = 24)					*PxGSS3* (N = 40)				
**Polymorphisms**		**syn_{JC}**: 56	**$nsyn_{JC}$**: 3				**syn_{JC}**: 86	**$nsyn_{JC}$**: 12				**syn_{JC}**: 97	**$nsyn_{JC}$**: 8			
**Substitutions**		**syn_{JC}**	**$nsyn_{JC}$**	**NI**	**χ^2_{JC}**	**P_{JC}**	**syn_{JC}**	**$nsyn_{JC}$**	**NI**	**χ^2_{JC}**	**P_{JC}**	**syn_{JC}**	**$nsyn_{JC}$**	**NI**	**χ^2_{JC}**	**P_{JC}**
**Sequence or ancestral sequence**	Model for reconstruction															
YcC	-	-	379.6	-	-	-	899.0	378.1	0.33	13.5	0.000238945	562.8	354.0	0.13	39.6	0.000000000
SulfC before separation of *Yponomeuta* and *Plutella* lineages	M0 (1 ω ratio)	467.1	278.8	0.09	25.1	0.000000555	424.2	277.5	0.21	27.7	0.000000139	347.2	249.2	0.11	44.9	0.000000000
	branch-site 1 ($\omega_2 = 1$)	442.5	287.8	0.08	27.6	0.000000146	388.7	287.9	0.19	33.1	0.000000009	280.7	250.8	0.09	56.9	0.000000000
	branch-site 1 ($\omega_2 > 1$)	422.4	283.5	0.08	28.6	0.000000089	372.8	283.5	0.18	34.3	0.000000005	264.8	246.6	0.09	59.3	0.000000000
	branch-site 2 ($\omega_2 = 1$)	507.2	298.2	0.09	24.7	0.000000671	445.0	298.3	0.21	28.9	0.000000075	367.8	255.0	0.12	43.2	0.000000000
	branch-site 2 ($\omega_2 > 1$)	431.9	289.2	0.08	28.6	0.000000091	395.3	289.3	0.19	32.6	0.000000011	393.8	260.6	0.12	41.1	0.000000000
	branch-site 3 ($\omega_2 = 1$)	442.5	287.8	0.08	27.6	0.000000146	388.7	287.9	0.19	33.1	0.000000009	280.7	250.8	0.09	56.9	0.000000000
	branch-site 3 ($\omega_2 > 1$)	422.4	283.5	0.08	28.6	0.000000089	372.8	283.5	0.18	34.3	0.000000005	264.8	246.6	0.09	59.3	0.000000000
SulfC before initial gene duplication in the *Plutella* lineage (*GSS1/2*, *GSS3*)	M0 (1 ω ratio)	124.3	109.7	0.06	34.8	0.000000004	124.2	110.8	0.16	36.2	0.000000002	52.2	86.9	0.05	75.7	0.000000000
	branch-site 1 ($\omega_2 = 1$)	160.2	147.7	0.06	37.6	0.000000001	158.0	148.9	0.15	40.8	0.000000000	30.9	55.0	0.05	68.0	0.000000000
	branch-site 1 ($\omega_2 > 1$)	164.2	150.1	0.06	37.4	0.000000001	162.0	151.4	0.15	40.5	0.000000000	30.9	55.0	0.05	68.0	0.000000000
	branch-site 2 ($\omega_2 = 1$)	59.9	127.2	0.03	71.2	0.000000000	65.1	129.6	0.07	77.1	0.000000000	91.1	72.4	0.10	41.0	0.000000000
	branch-site 2 ($\omega_2 > 1$)	12.3	97.0	0.01	111.2	0.000000000	18.7	100.4	0.03	111.8	0.000000000	165.9	102.8	0.13	34.0	0.000000000
	branch-site 3 ($\omega_2 = 1$)	160.2	147.7	0.06	37.6	0.000000001	158.0	148.9	0.15	40.8	0.000000000	30.9	55.0	0.05	68.0	0.000000000
	branch-site 3 ($\omega_2 > 1$)	164.2	150.1	0.06	37.4	0.000000001	162.0	151.4	0.15	40.5	0.000000000	30.9	55.0	0.05	68.0	0.000000000
GSS1/2 (before separation of *P. xylostella* and *P. australiana* lineages)	M0 (1 ω ratio)	1.0	6.0	0.01	34.6	0.000000004	7.1	12.1	0.08	25.3	0.000000485					
	branch-site 1 ($\omega_2 = 1$)	3.0	5.0	0.03	22.1	0.000002603	9.2	11.1	0.12	19.3	0.000011371					
	branch-site 1 ($\omega_2 > 1$)	3.0	5.0	0.03	22.1	0.000002603	9.2	11.1	0.12	19.3	0.000011371					
	branch-site 2 ($\omega_2 = 1$)	5.1	6.0	0.04	20.2	0.000006972	11.3	12.1	0.13	18.5	0.000016708					
	branch-site 2 ($\omega_2 > 1$)	5.1	6.0	0.04	20.2	0.000006972	11.3	12.1	0.13	18.5	0.000016708					
	branch-site 3 ($\omega_2 = 1$)	3.0	5.0	0.03	22.1	0.000002603	9.2	11.1	0.12	19.3	0.000011371					
	branch-site 3 ($\omega_2 > 1$)	3.0	5.0	0.03	22.1	0.000002603	9.2	11.1	0.12	19.3	0.000011371					
PxGSS1/2 (after separation of *P. xylostella* and *P. australiana* lineages)	M0 (1 ω ratio)	1.0	0.0	-	0.1	0.817922928	5.1	6.0	0.12	12.8	0.000344587					
	branch-site 1 ($\omega_2 = 1$)	1.0	0.0	-	-	-	5.1	6.0	0.12	12.8	0.000344587					
	branch-site 1 ($\omega_2 > 1$)	1.0	0.0	-	-	-	5.1	6.0	0.12	12.8	0.000344587					
	branch-site 2 ($\omega_2 = 1$)	1.0	0.0	-	-	-	5.1	6.0	0.12	12.8	0.000344587					
	branch-site 2 ($\omega_2 > 1$)	1.0	0.0	-	-	-	5.1	6.0	0.12	12.8	0.000344587					
	branch-site 3 ($\omega_2 = 1$)	1.0	0.0	-	-	-	5.1	6.0	0.12	12.8	0.000344587					
	branch-site 3 ($\omega_2 > 1$)	1.0	0.0	-	-	-	5.1	6.0	0.12	12.8	0.000344587					
GSS3 (before separation of *P. xylostella* and *P. australiana* lineages)	M0 (1 ω ratio)											35.5	5.0	0.58	0.8	0.365765569
	branch-site 1 ($\omega_2 = 1$)											30.9	7.0	0.36	3.5	0.059977742
	branch-site 1 ($\omega_2 > 1$)											30.9	7.0	0.36	3.5	0.059977742
	branch-site 2 ($\omega_2 = 1$)											22.0	6.0	0.30	4.5	0.033286162
	branch-site 2 ($\omega_2 > 1$)											22.0	6.0	0.30	4.5	0.033286162
	branch-site 3 ($\omega_2 = 1$)											30.9	7.0	0.36	3.5	0.059977742
	branch-site 3 ($\omega_2 > 1$)											30.9	7.0	0.36	3.5	0.059977742
PaGSS1/2	-	17.6	19.2	0.05	28.2	0.00000011	22.0	23.4	0.13	25.7	0.00000039					
PaGSS3	-											38.9	10.1	0.32	5.4	0.020287321

reconstructed nodes

SulfC before separation of *Yponomeuta* and *Plutella* lineages
(((((PxGSS1, PxGSS1), (PxGSS2, PxGSS2)), PaGSS1/2), ((PxGSS3, PxGSS3), PaGSS3)), YcC)

SulfC before initial gene duplication in the *Plutella* lineage (*GSS1/2*, *GSS3*)
((((PxGSS1, PxGSS1), (PxGSS2, PxGSS2)), PaGSS1/2), ((PxGSS3, PxGSS3), PaGSS3))

GSS1/2 (before separation of *P. xylostella* and *P. australiana* lineages)
(((PxGSS1, PxGSS1), (PxGSS2, PxGSS2)), PaGSS1/2)

PxGSS1/2 (after separation of *P. xylostella* and *P. australiana* lineages)
((PxGSS1, PxGSS1), (PxGSS2, PxGSS2))

GSS3 (before separatior ((PxGSS3, PxGSS3), PaGSS3))
((PxGSS3, PxGSS3), PaGSS3))

branch-site models

branch-site 1 ($\omega_2 = 1$)	((((PxGSS1, PxGSS1), (PxGSS2, PxGSS2)), PaGSS1/2), ((PxGSS3, PxGSS3), PaGSS3)) #1
branch-site 1 ($\omega_2 > 1$)	((((PxGSS1, PxGSS1), (PxGSS2, PxGSS2)), PaGSS1/2), ((PxGSS3, PxGSS3), PaGSS3)) #1
branch-site 2 ($\omega_2 = 1$)	(((PxGSS1, PxGSS1), (PxGSS2, PxGSS2)), PaGSS1/2) #1
branch-site 2 ($\omega_2 > 1$)	(((PxGSS1, PxGSS1), (PxGSS2, PxGSS2)), PaGSS1/2) #1
branch-site 3 ($\omega_2 = 1$)	((PxGSS3, PxGSS3), PaGSS3)) #1
branch-site 3 ($\omega_2 > 1$)	((PxGSS3, PxGSS3), PaGSS3)) #1

Table S7. Primers used in this study.

Name	Sequence (5'→3')	Usage
PXY_GSS3FpIB	GTGATGGGTCGGCTAGTGCAGATC	expression
PXY_GSS3RpIB	AGCGGTGTAAGGCTCGAGGTC	expression
PXY_SulfDpIB	GAAATGGTGTACAAATGGGCGC	expression
PXY_SulfDpIB	GAAAACATTTTGTAAAACGCATATAAACTG	expression
PXY_GSS1FpIB	GTGATGGCGATTCTGCATCAAGCTG	expression
PXY_GSS1RpIB	TTCGGAGTCGGAGTAGGGTTC	expression
PXY_GSS2FpIB	AGCATGGCAATTCTGCATCAAGTAGTGGTGATTCTG	expression
PXY_GSS2RpIB	TTCAGAGTCAGAGTAGGGTTCAACAGAG	expression
BMO_C1FpIB	GAAATGGTCGTGTTTCGAGTTTTGCTG	expression
BMO_C1RpIB	ATCAATAAATGTATCCCACGTATTGTTATG	expression
YCA_CFpIB	GAAATGGTTTTGCTAGCGGCCTTTTCG	expression
YCA_CRpIB	TTTTTTATTTATCTGATTCTTTGACTTAATGG	expression
PXY_GSS3Fq	CTGAGGTCGCTGAAGGAAAC	RT-qPCR
PXY_GSS3Rq	TGATGTCGTACAGGCACCAT	RT-qPCR
PXY_SulfDFq	TACCTCAAGGAGCTCGGCTA	RT-qPCR
PXY_SulfDRq	AATGCTCTCACCACCTGACC	RT-qPCR
PXY_GSS1Fq	CATCAAGCTGTGGTGCTCCTC	RT-qPCR
PXY_GSS1Rq	CTGCCGTCAAGCTGCTCC	RT-qPCR
PXY_GSS2Fq	CTGCATCAAGTAGTGGTGATTCTG	RT-qPCR
PXY_GSS2Rq	GCTTGCCGTCAAGCCTCGAT	RT-qPCR
PXY_RPS18Fq	ATGTCGCTCGTAATACCGGAC	RT-qPCR
PXY_RPS18Rq	CAGTAGTGACGCATGCCTCG	RT-qPCR
